# Tunable Magnetic Order in Fe-Mg Codoped Montmorillonite Nanoclay Interfaced with Amino Acids

**DOI:** 10.1021/acsomega.4c06483

**Published:** 2025-01-10

**Authors:** Dinesh Thapa, Steven Westra, Victoria Oas, Dmitri Kilin, Svetlana Kilina

**Affiliations:** † Department of Mathematics and Physics, Thomas More University, Crestview Hills, Kentucky 41017, United States; ‡ Department of Chemistry and Biochemistry, North Dakota State University, Fargo, North Dakota 58108, United States

## Abstract

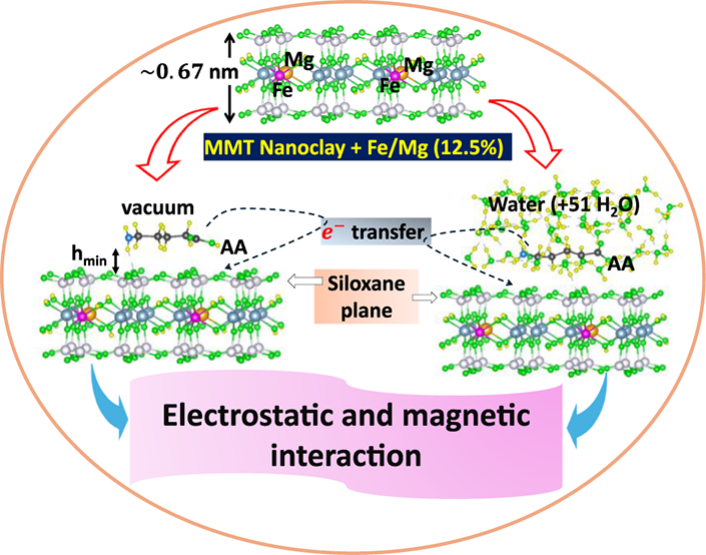

This study elucidates the sensitivity of the spin magnetic moment of the Fe–Mg codoped montmorillonite (MMT) nanoclay to its interactions with three unnatural amino acids (AAs): 5-aminovaleric acid, 2-aminopimelic acid, and DL-2-aminocaprylic acid, in the presence and absence of an aqueous environment. These AAs are known as intercalating agents for MMT clay, providing the formation of the nanoplates. Using spin-polarized density functional theory (SP-DFT), the magnetic moment and its tunability to the position of Fe and Mg impurities in the MMT nanoclay crystal lattice, along with the alignment of AA molecules on the nanoclay surface, have been investigated. There is substantial charge transfer between the AA molecule (a donor) and the MMT nanoclay (an acceptor), indicating their strong electrostatic interaction. Moreover, it is found that AA molecules stabilize Fe(II) and prevent its oxidation to Fe(III) through strong interactions with the nanoclay, highlighting the significance of clay–amino acid interactions. The calculations predict the possible transition in magnetic orders (ferromagnetic, antiferromagnetic, and ferrimagnetic) governed by interactions between the MMT nanoclay and the AA molecules in the vacuum and aqueous medium. The significant magnetic exchange coupling observed in some of the nanoclay models, in the presence of an aqueous medium, suggests a unique property of quantum ferrofluids. These findings indicate promising applications of these materials in biomedicine and bioengineering, particularly in the areas requiring an electromagnetic response, such as magnetic resonance and magneto-optical imaging, magnetic drug targeting, hyperthermia cancer treatment, magnetic separation, and magneto-mechanical sensors.

## Introduction

Montmorillonite (MMT) is a naturally occurring smectite group of clay minerals, which possess a unique set of properties, including but not limited to strong adsorption capacity, biocompatibility, antimicrobial activity, ion-exchange, and swelling ability.^1−4^ The clay minerals, such as MMT, have the ability to adsorb various organic molecules, including amino acids and nucleic acids, in the natural environment. This property benefits clay applications in soil ecosystems, biochemical evolution, biosensing, genetic information storage, protein fractionation, pharmaceutical practices, and enzyme immobilization.^5−7^ The adsorption mechanism of the amino acids (AA) depends on the type and properties of the clay minerals, such as cation exchange capacity, surface charge density, surface area, and degree of expansibility.

Several studies underscore the superior adsorptive properties of MMT clay over kaolinite and other clays. Thus, it has been found that in phosphate buffer, the adsorption capacity of the toxic protein from *Bacillus thuringiensis* on the MMT nanoclay was much higher than that on kaolinite.^8^ Similarly, it has been reported that MMT clay bound higher amounts of human serum albumin (HSA), β-glucuronidase (GUS), and Cry3Bb1 than kaolinite under the same experimental conditions.^9^ It has also been demonstrated that MMT clay can be used as a promising material in tissue engineering because of its improved biocompatibility when modified with unnatural amino acids, such as valeric acid.^10,11^ In particular, MMT clay intercalated into nanoplates by AA molecules and blended with chitosan/polygalacturonic acid (ChiPgA) has been used to prepare composite scaffolds, which act as a precursor for the proliferation of human osteoblast cells, satisfying the important requirements for tissue engineering applications.^10^

In addition to the chemical composition of clay minerals, the geometrical structure, molecular size, and physical and chemical properties of the amino acid molecules also play a pivotal role in determining the possible adsorption sites on the clay minerals. The adsorption mechanism also relies on the electrostatic interaction, hydrogen bonding, exchange of surface ligands, formation of cation bridges, dielectric constant of the medium, etc., showing the complexity of clay-molecule interactions and the need for further investigations to fully understand and predict these processes.

**Figure 1:**
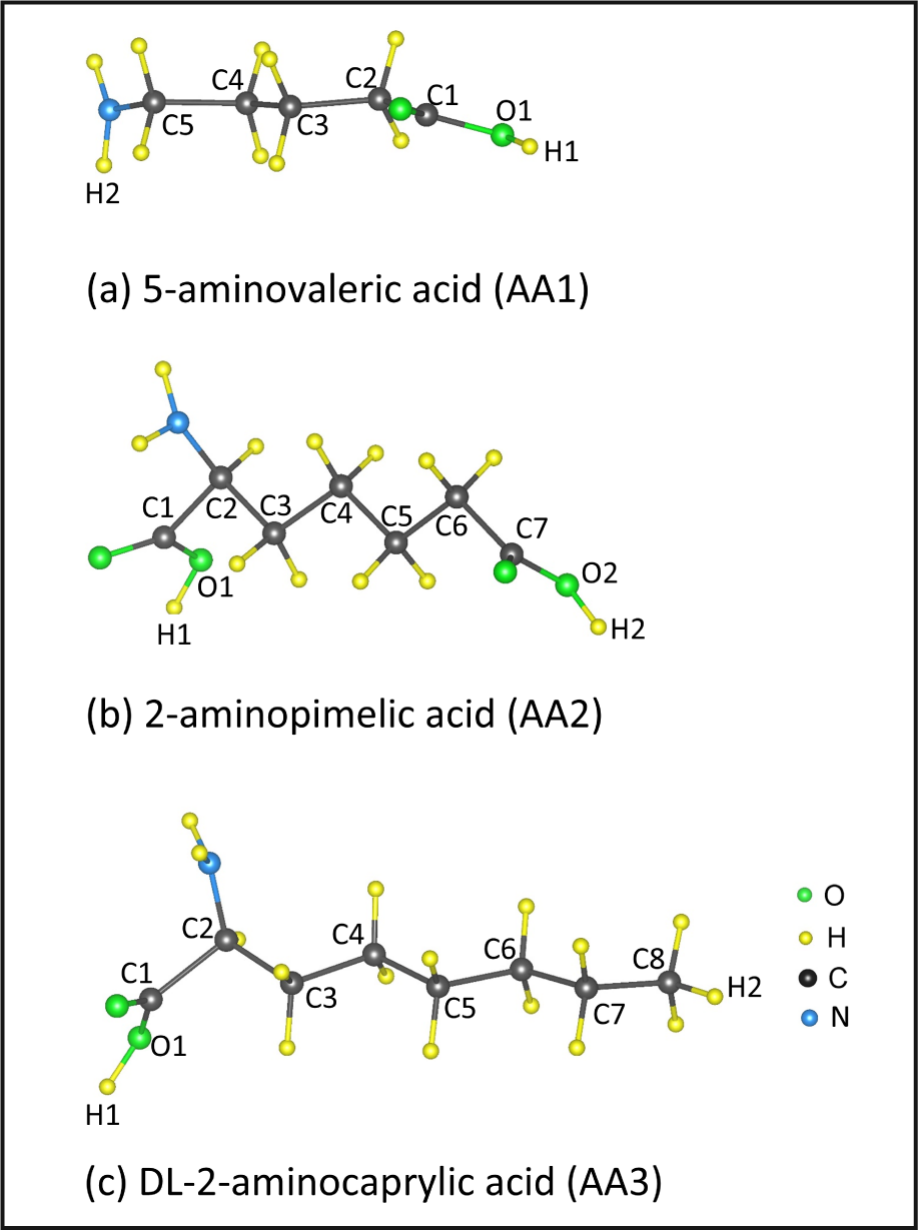
Optimized geometries of three different types of amino acid (AA) molecules used as adsorbates on the MMT nanoclay surface.

In this paper, a computational study was conducted on the adsorption process between MMT nanoclays and three unnatural amino acids (AAs), such as 5-aminovaleric acid (AA1), 2-aminopimelic acid (AA2), and DL-2-aminocaprylic acid (AA3), with molecular weights of 117.15, 175.18, and 159.23 g/mol, respectively. The optimized geometries of the isolated AA molecules are shown in Figure 1. These AA molecules have been experimentally shown to be efficient intercalating agents for producing MMT nanoclays of about 1.0 nm in thickness.^10,11^ Using spin-polarized density functional theory (SP-DFT), we have gained atomistic insights into the changes in the electrostatic and magnetic properties of Fe–Mg codoped MMT nanoclay induced by adsorbed AA1, AA2, and AA3 molecules, both in the vacuum and in the aqueous medium.

Previous studies^12^ have shown that all clay minerals contain divalent or trivalent impurities, such as Fe, Cr, Mg, Ca, etc. The most common impurities for MMT are Mg and Fe ions, replacing Al in the octahedral sheets. Introducing Fe, Mn, or V ions with an unpaired number of electrons in their d-shell into two-dimensional (2D) insulating or semiconducting nanosheets has been observed to induce magnetization, resulting in the system becoming either a dilute magnetic semiconductor or a semimetal, making it suitable for various spintronic applications.^13−15^ As such, the interaction between Fe(II)-containing MMT nanoclays and their surroundings is expected to alter the magnetic properties of the nanoclay while significantly influencing various reactions important for environmental and bioapplications.^16^

Substituents such as Fe(II) and Mg(II) for Al(III) lead to a negative excess charge, which is typically counterbalanced by alkali and alkaline earth metal cations situated between the layers in bulk MMT clay.^17^ Cationic alkyl-ammonium and nonionic ethoxylated amine^18^ are used to exfoliate layers, resulting in MMT nanoclays. Joint experimental and molecular modeling studies demonstrate strong stabilization of protonated aminovaleric acid on the MMT surface with octahedral substituents of Fe(II) and Mg(II) ions, accompanied by the replacement of Na^+^ cations from the intercalated modified MMT surface.^4^ However, the chemistry of such amino acids is complicated by the fact that the −NH_2_ group is a base, and the −COOH group is an acid. Therefore, in aqueous solution, the protonation of the −NH_2_ group and the deprotonation of the −COOH group are in dynamic equilibrium, resulting in both positively/negatively charged and neutral molecules. The cation exchange with the MMT surface during layer exfoliation may change this equilibrium toward neutral species.

Our calculations are set up to explore whether the unbalanced charge on the Fe/Mg doped MMT nanoclay and neutral AA molecules provide strong AA–nanoclay interactions and how they affect the electronic and magnetic properties. To get atomistic insights into the clay–molecule interactions and to elucidate the role of magnetic impurities, the slab structures of the MMT nanoclay were modeled with four Al(III) ions on the octahedral sites being cosubstituted with two Fe(II) and two Mg(II) ions. Each of the AA molecules under study is then adsorbed on the nanoclay surface. The clay–AA interaction, or binding energy (*E*_*b*_), changes in the charge density (Δρ), and the magnetization density, and the ground state magnetic order of the nanoclay/AA composites both in the vacuum and in the aqueous medium are simulated using the SP-DFT+U method with included van der Waals corrections. The calculations reveal that the intricate nature of the interactions between the MMT nanoclay and the AA molecules provides valuable information for understanding the adsorption mechanisms and their impacts on the electrostatic and magnetic properties, critical for creating nanosized materials for tissue engineering and various biomedical applications.

## Computational Methods

**Figure 2:**
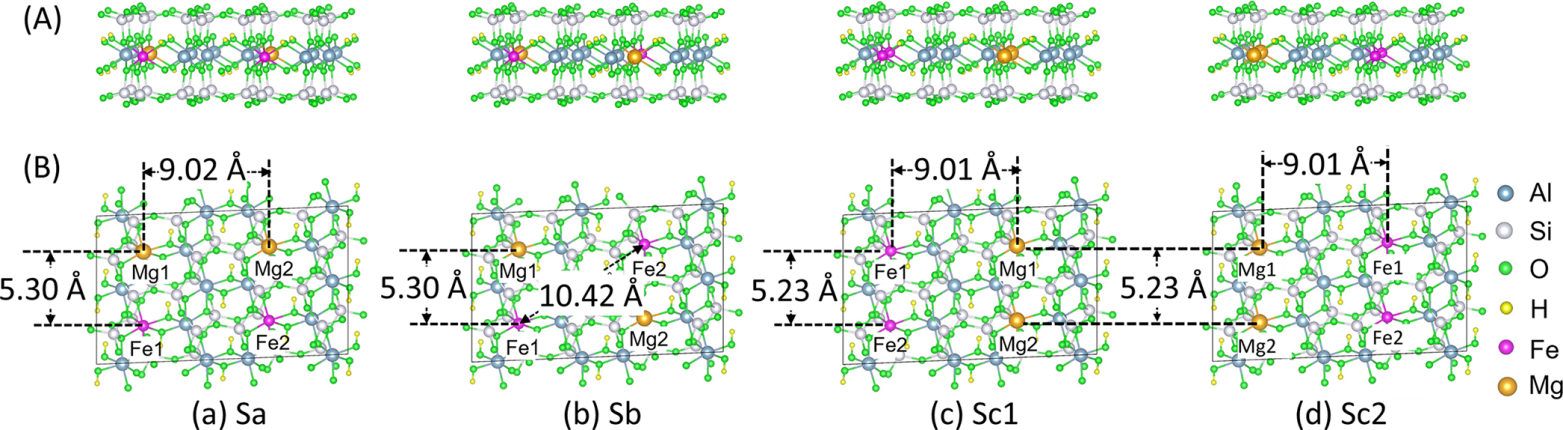
Optimized geometrical structures of four different nanoclay surfaces Sa, Sb, Sc1, and Sc2, respectively, with (A) the side view along the (100) plane slightly rotated along the *ab* invariant to the *z-*axis. (B) The top view along the (001) plane showing the spatial positions of dopant atoms, Fe(II) and Mg(II) constituting the four vertices of a parallelogram. The solid gray line represents the size of the supercell.

First-principle calculations based on DFT were performed using the projector augmented wave (PAW) method^19^ within the generalized gradient approximation (GGA) as employed in the Vienna Ab-Initio Simulation Package (VASP).^20,21^ The calculations employed the Perdew-Burcke-Ernzerhof (PBE) exchange-correlation functional within GGA.^22^ The plane wave energy cutoff of 520 eV was used for all calculations, ensuring adequate accuracy. Because of the computational cost, resources, and time constraints for geometrical relaxations, we constructed the supercell size of 2 × 2 × 1 from the relaxed primitive and pristine unit cell of the bulk, to construct nanoclay or nanoslab models, as shown in Figure S1 and Figure 2, respectively. This supercell size is sufficient to minimize the interactions of the impurity atoms with their neighboring unit cells. A sufficiently large vacuum length of ∼11.2 Å was set along the *z*-direction in the MMT nanoclay model in order to provide enough space for adsorbed AA molecules and safely avoid the spurious interaction between the adjacent layers due to periodic boundary conditions (PBC). This size of the computational cell of the MMT nanoclay model has been shown to be reasonable in previous calculations for similar systems.^23^

All the geometrical structures of the MMT clay, including pristine and Fe–Mg codoped nanoclay (nanoslab) models of monolayer thickness, were fully optimized, including the lattice vectors and atom positions. The doped MMT nanoclays were interfaced with one of the unnatural AA molecules (AA1, AA2, and AA3), as shown in Figure S2 and allowed to fully relax with their atom positions both in the vacuum and in the aqueous medium. The explicit solvation model (ESM) was employed, introducing 51.0 water molecules into the vacuum region in the simulation cell to mimic the aqueous medium in the system, as described in the previous works.^24,25^

Optimization of all geometries was performed until the total energy was converged numerically to less than 1.0 × 10^–5^ eV per unit cell and the force on each atom was less than 10^–2^ eV/Å. The geometry optimization was performed using the Monkhorst-Pack k-point mesh of 4 × 2 × 4 and 4 × 2 × 1 for the bulk and slab models, respectively. The optB86b-vdW exchange functional was employed to correct the van der Waals (vdW) interactions between the neighboring layers of MMT nanoclay mineral, along with the accurate description of the lattice parameters and the clay–molecule interaction energies.^26−28^ The visualization of all calculated structures was produced using VESTA software.^29^

All the calculations were collinear and spin-polarized for the magnetic system, except for the nonmagnetic system where spin polarization was switched off. In collinear calculations, the total energy of the system is invariant with the rotation of the spins relative to the crystallographic cell. Thus, the magnetic moments expressed in this paper are purely scalar quantities.^30^ To perform the magnetic order calculations, two possible configurations were chosen for each magnetic order of ferromagnetism (FM), antiferromagnetism (AFM), and ferrimagnetism (FEM), as shown in Figure S3. The lowest energy configuration of the two configurations was chosen to predict a more stable magnetic order. The symmetry of the systems was switched off entirely while performing magnetic calculations to achieve the desired magnetic order. In some of the AFM and FEM configurations, where it is hard to achieve the expected magnetic order, especially for AFM configurations, the total magnetic moment was constrained to zero using the option NUPDOWN as available in the VASP input setting.

### DFT+U Method

On account of the strongly correlated electronic systems, we employed the PBE functional with a Hubbard correction term, *U*_eff_, within Dudarev’s approach.^31^ The DFT+U total energy is given by eq 1:
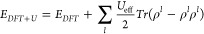
1where ρ^*l*^ is the atomic orbital occupation matrix. The inclusion of the Hubbard correction is known to be significant to account for the underestimated electronic interactions in transition metal elements. It adds a semi-empirically tuned numerical parameter, *U*_eff_ = *U* – *J*, with *U* and *J* representing the on-site Coulomb term and the site exchange term, respectively. In the present work, *U*_eff_ = 4.0 eV with *J* = 1.0 eV was chosen for the localized *d*-orbitals of the Fe(II), throughout the paper unless otherwise stated. The *U*_eff_ = 4.0 eV for the Fe atom underpins the accuracy of calculated electronic and magnetic properties, consistent with the experiments as mentioned in previous works related to Fe-doped MMT nanoclays and similar systems.^32−38^ To verify the accuracy of the results, we also tested *U*_eff_ = 3.0 eV as a correction parameter for the localized *d*-orbitals of the Fe(II) atom in some of the selected nanoclay models, Sa+(AA1, and AA2) in the vacuum. The effective *U*_eff_ values for all other atoms Al, Mg, Si, O, C, N, and H were set to zero.

## Results and Discussions

In this section, the calculated results on the optimized geometries of the Fe–Mg codoped MMT nanoclay models with adsorbed AA molecules, surface energy (E_γ_) and binding energy (*E*_*b*_), minimum vertical equilibrium distances (*h*_min_) between adsorbed AA molecules and the nanoclay surface, charge density differences (Δρ), and finally the ground state magnetic order in different nanoclay models are discussed in detail.

### Structural Characteristics of Pristine and Doped MMT Nanoclay

The pristine MMT nanoclay with the empirical chemical formula Al_2_Si_4_O_10_(OH)_*2*_.*n*H_2_O is a member of the smectite group. It is a 2:1 clay, which means that it has two tetrahedral sheets of silica sandwiching a central octahedral sheet of alumina in 6-fold coordination with hydroxyl (−OH) groups and oxygens from the tetrahedral sheets. The optimized geometries and the structural parameters of the bulk and a single nanoclay sheet of the pristine MMT are shown in Figure S1. The two adjacent layers in the MMT clay interact with each other by a weak vdW forces.^39^ From the numerical results, a large interlayer distance in the bulk MMT clay, ∼2.442 Å, signifies a weak interlayer interactaion . Due to this weak interaction, the adjacent layers can be easily intercalated or exfoliated into a monolayer sheet of a nanoclay, enhancing surface interaction of MMT with the organic molecules and polymers.^40^

The optimized lattice parameters for the primitive bulk MMT clay structure are *a* = 5.180 Å, *b* = 8.948 Å, *c* = 9.269 Å, α = 80.93°, β = 83.82°, and γ = 90.06°, which are consistent with previously reported results.^41^ Also, the optimized lattice parameters for a primitive monolayer sheet are *a* = 5.192 Å, *b* = 8.963 Å, α = 80.77°, β = 83.61°, and γ = 90.00°. Both the bulk and the monolayer sheets of the MMT clay exhibit the same crystallographic symmetry of *P*1(*no*.1). From the electronic structure calculations, both the pristine bulk and a single nanosheet of the MMT clay act as insulators with a substantial band gap of 5.56 and 5.69 eV, respectively, when calculated using the PBE functional.

The doping of the MMT nanoclay is introduced by substituting aluminum (Al(III)) at four different octahedral sites with two different foreign ions, iron (Fe(II)) and magnesium (Mg(II)), so that the dopant ions act as the vertices of the parallelogram, as shown in Figure 2. The impurity concentration of Fe(II) and Mg(II) in the MMT nanoclay is 12.5% each. This concentration agrees with the experimentally estimated 13% to 24% octahedral iron by weight found in the natural iron-rich MMT clays.^42,43^ We have designed four different Fe–Mg codoped MMT nanoclay models Sa, Sb, Sc1, and Sc2 based on the spatial positions of Fe(II) and Mg(II), as illustrated in Figure 2. All nanoclay structures have the empirical chemical formula (FeMg)_0.25_Al_1.5_Si_4_O_10_(OH)_2_.*n*H_2_O, where *n* = 51.0 represents the number of water molecules introduced into the computational cell. The nanoclay structures are characterized by the monolayer thickness of ∼0.67 nm with a sufficiently large vacuum length of 11.2 Å along the *z*-axis and a large surface area of 180.76 Å^2^ providing enough space for the adsorption of AA molecules.

The nanoclay models are distinct from each other by the Fe–Fe or Mg–Mg separation along the longest side (9.02 Å) in nanoclay Sa, along the diagonal (10.42 Å) in nanoclay Sb, and along the shortest side (5.23 Å) in slab Sc1 or Sc2 of the computational cell. The variations in the Fe–Fe distance are anticipated to have distinct impacts on the magnetic properties of the nanoclay with adsorbed AA molecules. The optimized shortest Fe–Fe, Fe–Mg, and Mg–Mg distances in the four different nanoclay models are listed in Figure 2B. Note that the nanoclay models Sc1 and Sc2 are periodically identical in their crystal lattice, while the difference is introduced by the alignment of the AA molecule on the nanoclay surface with respect to the Fe–Fe position.

### Calculation of Surface Energy (γ)

The surface energy (E_γ_) of the symmetric and stoichiometric nanoclay models along the easily cleavable surface (001) plane was calculated using eq 2 following the procedure explained in the paper by Tian et al.^44^
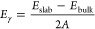
2where *E*_slab_ is the total energy of a single-layer nanoclay model, *E*_bulk_ is the total energy of the bulk structure with the same number of atoms as that in the nanoclay of a supercell size of 2 × 2 × 1, and *A* is the surface area of the nanoclay model. The calculated surface energies for the Fe(II)–Mg(II) codoped MMT nanoclay models Sa, Sb, and Sc (Sc1 or Sc2) are 152.72 mJ/m^2^, 153.42 mJ/m^2^, and 175.34 mJ/m^2^, respectively. The purpose of choosing the surface normal to the (001) plane in the MMT clay structure is its possibility to cleave along the x-y plane, due to the weak interlayer van der Waals interaction along the *z*-direction. The lower values of the calculated surface energy suggest that the Fe–Mg-codoped MMT nanoclays are easier to form than Li, Na, K, Rb, and Cs containing MMT nanoclays, whose reported surface energies are 391 mJ/m^2^, 424 mJ/m^2^, 532 mJ/m^2^ 661 mJ/m^2^, and 838 mJ/m^2^, respectively.^45^

### Calculation of Interaction Energy between MMT Nanoclay and Adsorbed AA Molecules

The interaction energy between MMT nanoclay and adsorbed AA molecules has been studied in terms of binding energy (*E*_*b*_) of the AA molecules to the surface of MMT nanoclay. The binding energy that represents the binding affinity for the adsorption of three different AA molecules on the nanoclay surface in the vacuum, as well as in the aqueous medium, was calculated using eqs 3 and 4, respectively.
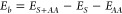
3
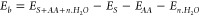
4where *E_S+AA_* and  are the total energies of the nanoclay with the adsorbed AA molecule in the vacuum and in the aqueous medium, respectively. Here, the values of *E*_*S+AA*_ and  are so chosen that they represent the lowest stable configuration out of four magnetic orders (FM, AFM, FEM, and NM) in nanoclay models. *E*_*S*_, *E*_*AA*_, and  are the relaxed energies of the isolated Fe–Mg codoped nanoclay (S), the amino acid (AA), and *n* number of water molecules, respectively, calculated at the same size of computational cell. Here, the free standing aqueous surface comprising n number of water molecules is found to achieve minimum energy per water molecule compared to an isolated water molecule within the same box size. Based on the explicit solvent model (ESM), the number of water molecules is determined to cover the free volume (*V*_free_) in the vacuum region within the supercell, excluding the volume occupied by the nanoclay and the adsorbed AA molecule. From there, one can get a density of water approximately equal to that of pure water (1.0 g/cm^3^). Thus, the estimated value of *n* in eq 4 is calculated using eq 5:
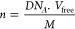
5where *D* = 1.0 g/cm^3^ is the density of pure water, *N_A_* is Avogadro’s number, *M* = 18.0 g/mol is the molar mass of water, *V*_free_ = *V*_*SC*_–*V*_*AA*_–*V*_*S*_ is the free volume in the vacuum region excluding the volume of amino acid (*V*_*AA*_) and the nanoclay (*V*_*S*_), and *V*_*SC*_ is the total volume of the supercell of size, 2 × 2 × 1. Based on this assumption, the value of *n* = 51 approximately satisfies the requirement to cover the vacuum region in the MMT nanoclay models.

**Figure 3:**
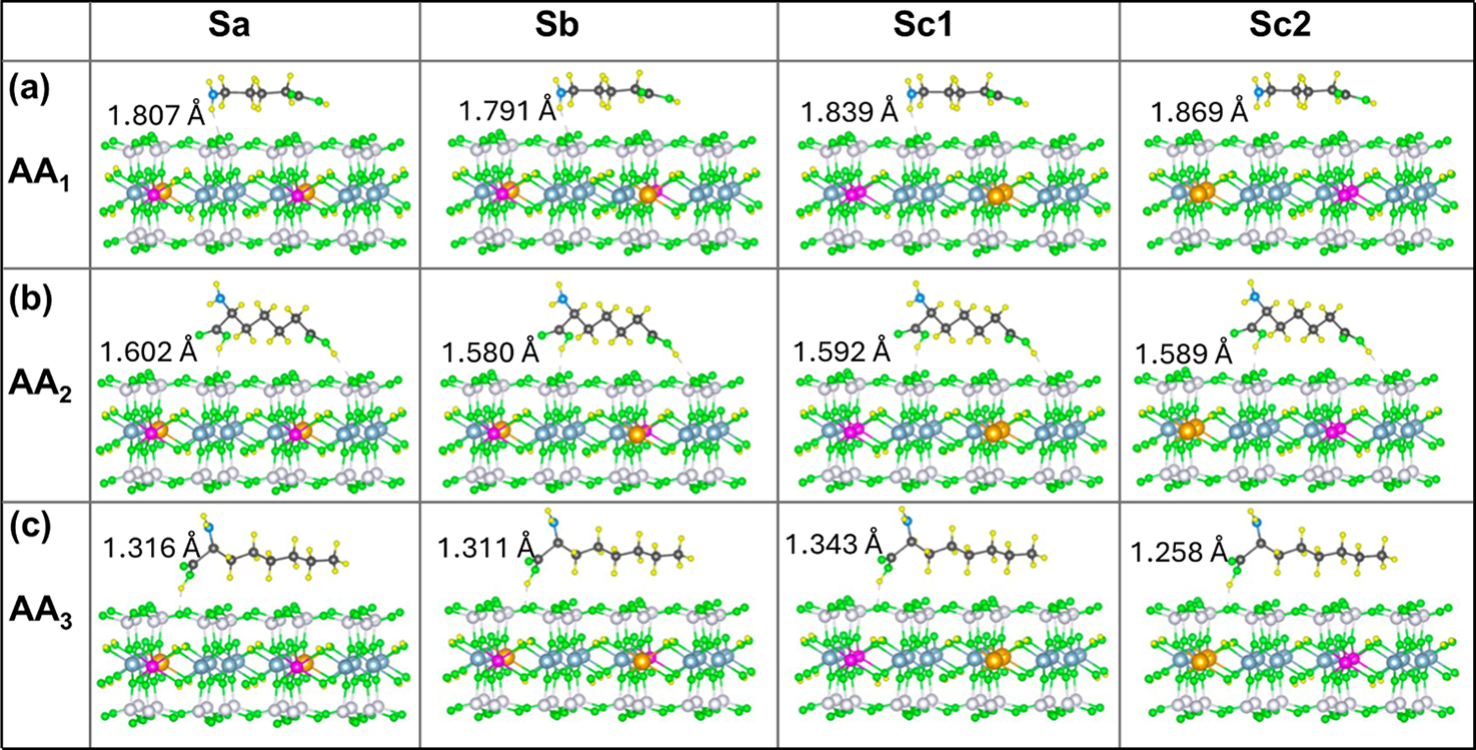
Optimized geometries of adsorbed amino acids (a) AA1, (b) AA2, and (c) AA3 on different nanoclay models Sa, Sb, Sc1, and Sc2 in the vacuum. The numbers in the inset represent the minimum vertical distance (*h*_min_) between the AA molecule and the nanoclay surface.

Within this definition, the negative values of the binding energies correspond to the thermodynamically stable configurations. Thus, the obtained negative values of the binding energies in all cases calculated in the vacuum and in the aqueous medium (Table S1) indicate that the adsorption of all considered AA molecules on the nanoclay surface is feasible via an exothermic process. In the vacuum, the degree of the interaction between the AA molecule and the MMT nanoclay depends considerably on the position of the electron-donating (−NH_2_) and electron-withdrawing (−COOH) groups of the molecules lying toward or away from the dopant ions, as can be seen in Figure 3.

**Figure 4:**
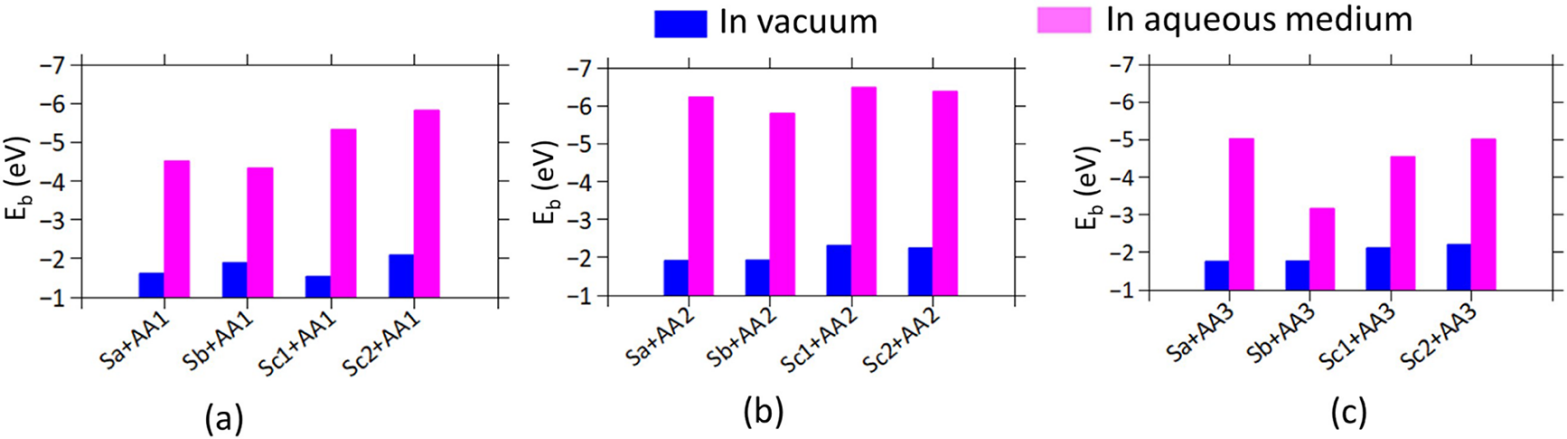
Bar diagram representing interaction or binding energy (*E_b_*) due to the adsorption of amino acids (a) AA1, (b) AA2, and (c) AA3 on the four different nanoclay models,both in the vacuum and in the aqueous medium.

Thus, for AA1 adsorption in the vacuum, the strongest interaction with the nanoclay occurs when the −NH_2_ group is closest to the slab toward the shortest Fe–Mg separation while the Fe–Fe distance is maximized (Sb model), or when the −NH_2_ group is near the nanoclay toward the shortest Mg–Mg separation while the −COOH group is positioned toward the shortest Fe–Fe separation (Sc2 model), as shown in Figure 3a. For AA2, a stronger interaction occurs when one −COOH group faces the shortest Fe–Fe separation and the other faces the shortest Mg–Mg separation in the Sc1 and Sc2 nanoclay models, Figure 3b. Similarly, for AA3, a stronger interaction occurs when the −COOH group faces either the shortest Fe–Fe or the shortest Mg–Mg separation in the Sc1 and Sc2 nanoclay, respectively, as shown in Figure 3c. Overall, all AA molecules show the strongest binding affinity of 2.1–2.3 eV in absolute values (Table S1), when the −COOH (AA2 and AA3) or −NH_2_ (AA1) group closest to the surface faces the shortest Mg–Mg separation in the Sc1 or Sc2 nanoclay, which also have the shortest Fe–Fe distance. Among the most stable conformations, AA2 and AA3 interact slightly more strongly than AA1, as can be seen in Figure 4 (blue bars) and Table S1.

The preferred interactions of AA molecules with the nanoclay Sc1 or Sc2 are also observed in the aqueous medium (Table S1). However, the binding affinity of all AA molecules is significantly enhanced in the presence of an aqueous medium (Figure 4, magneta bars). Compared to the vacuum cases, the AA–nanoclay interaction energy not only depends on the alignment of the electron-donating and withdrawing groups of the AA molecule but also on the neighboring water molecules contributing additional dipole–dipole interactions with both the AA and the nanoclay surface.^46^ This highlights the significant role of the aqueous environment in strengthening the AA-nanoclay interactions compared to the vacuum cases and underscores the importance of considering the environmental factors in studying such interactions.

### Determination of the Equilibrium Nanoclay–AA Distance

**Figure 5:**
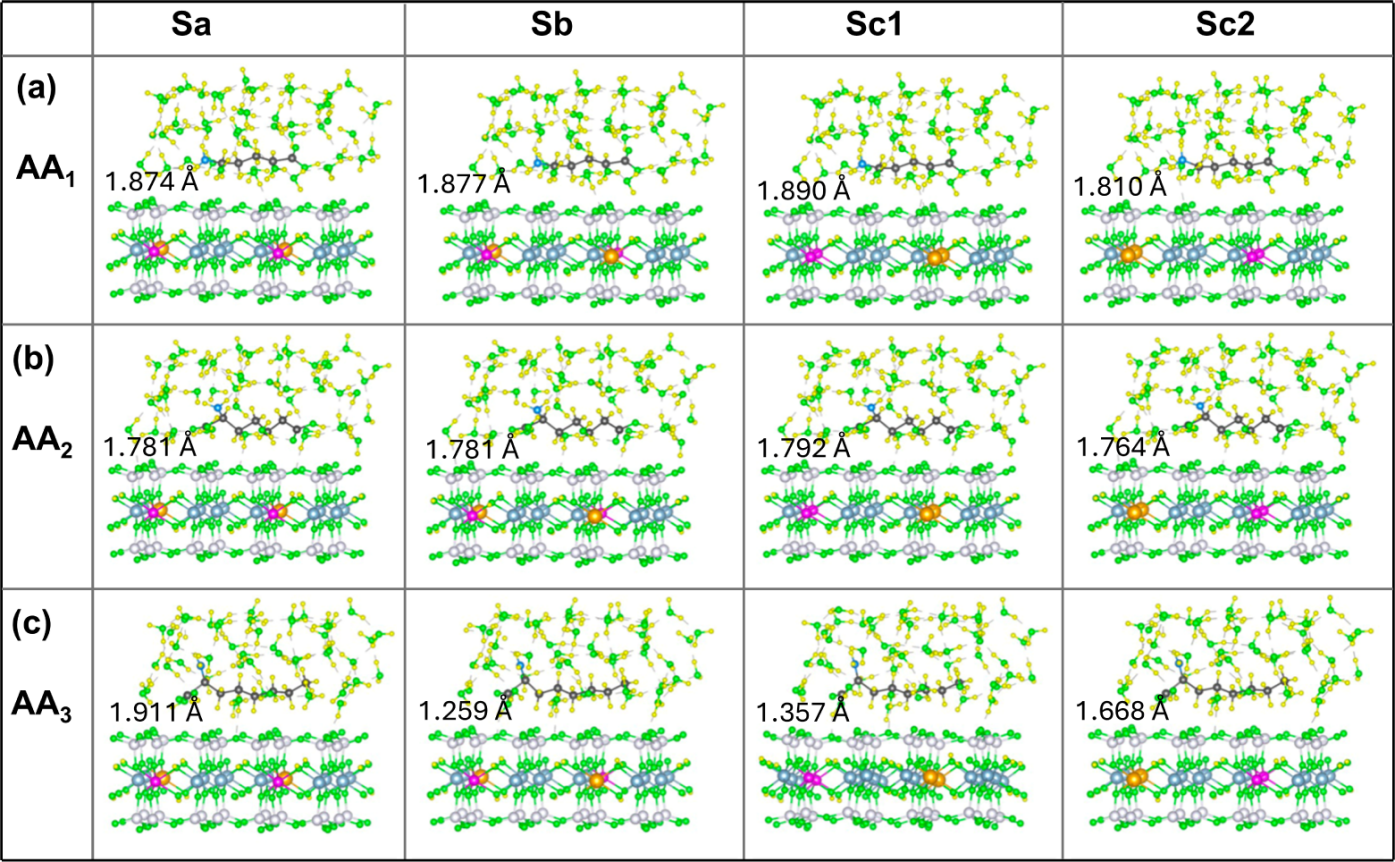
Optimized structures of adsorbed amino acids (a) AA1, (b) AA2, and (c) AA3 on different nanoclay models Sa, Sb, Sc1, and Sc2 in the aqueous medium. The numbers in the inset represent the equilibrium vertical distance (*h*_min_) between the AA molecule and the nanoclay surface.

The equilibrium adsorption distance between the nanoclay surface and the AA molecule is calculated in terms of minimum adsorption height (*h*_min_), which is the vertical distance between the atom of the AA molecule nearest to the surface of the nanoclay, as illustrated in Figure 3 for vacuum and Figure 5 for the aqueous medium. It is found that the value of *h*_min_ decreases from 1.8 to 1.3 Å while going from AA1 to AA3 for all nanoclays in the vacuum. This trend, with slightly increased values of *h*_min_ is noted in the aqueous medium except for the Sa+AA3 case, where the *h*_min_ is comparable to those of Sa+AA1. The equilibrium adsorption distance (*h*_min_) and the smallest vertical distance to the individual atoms, nitrogen (N), oxygen (O), hydrogen (H), and carbon (C) of the AA molecule, from the nanoclay surface in the vacuum and in the aqueous medium are represented in Tables S2–S4. The equilibrium distance and vertical positions of atoms vary between vacuum and aqueous environments. These variations, along with a slight increase in *h*_min_ in water, are physically due to the random Brownian movement of the AA molecules in the water, which competes with a strong dipole–dipole interaction between the AA molecule and the nanoclay surface. Additionally, this interaction involves electronegative N and O atoms of the AA chain and O atoms of the nanoclay surface with the H atoms of water molecules, forming hydrogen bonds. These hydrogen bonds increase the nanoclay–AA interaction energy in the water compared to the vacuum.

### Calculation of the Charge Density Difference (Δρ)

**Figure 6:**
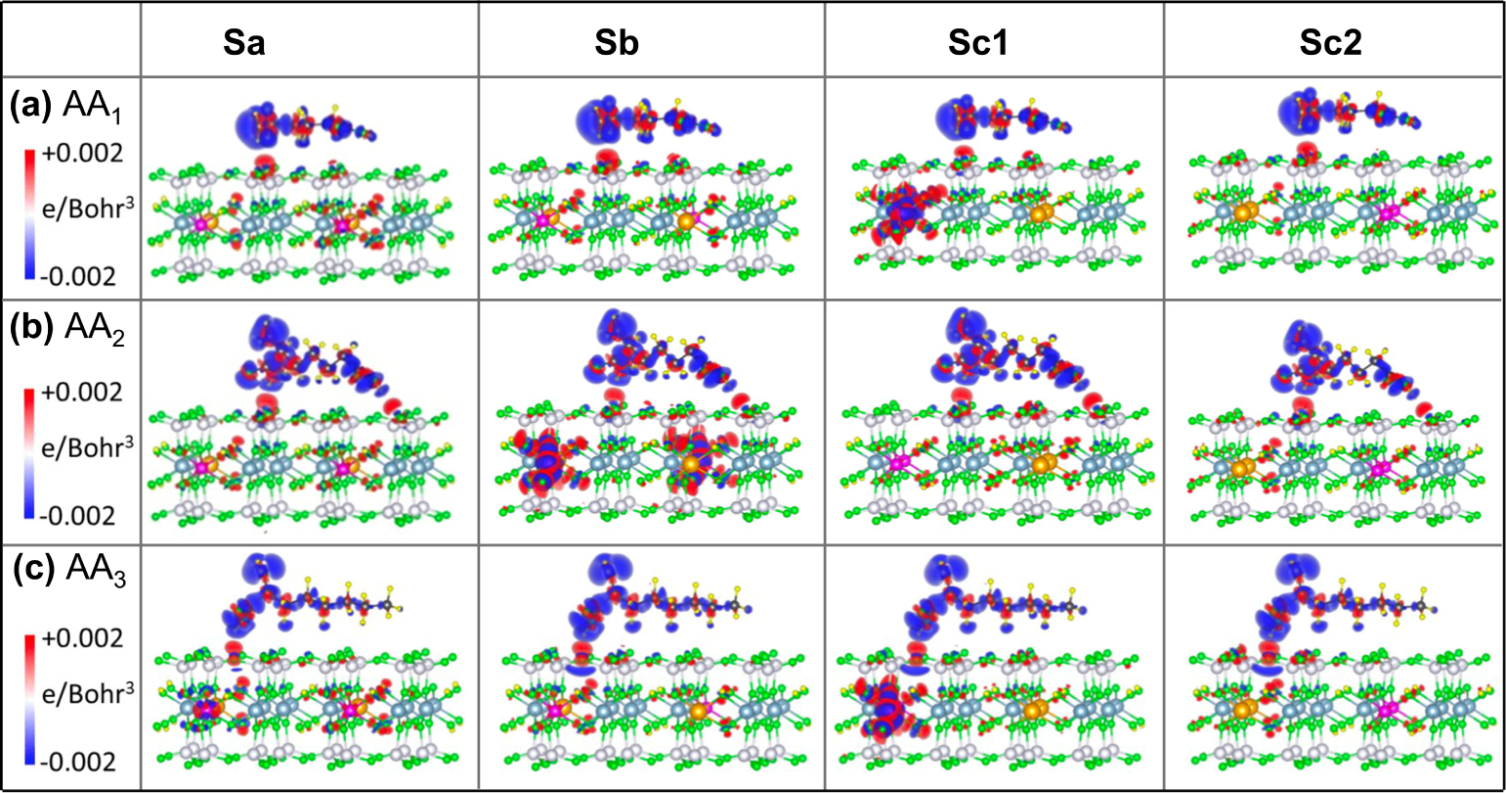
3D iso-surface plots of charge density difference (Δρ) caused by the adsorption of the AA molecules (a) AA1, (b) AA2, and (c) AA3 on different nanoclay models Sa, Sb, Sc1, and Sc2 in the vacuum. The iso-surface value ranges from −0.002 e/Bohr^3^ to +0.002 e/Bohr^3^, with the negative values (blue) representing the regions of charge depletion and the positive values (red) corresponding to the charge accumulation.

**Figure 7:**
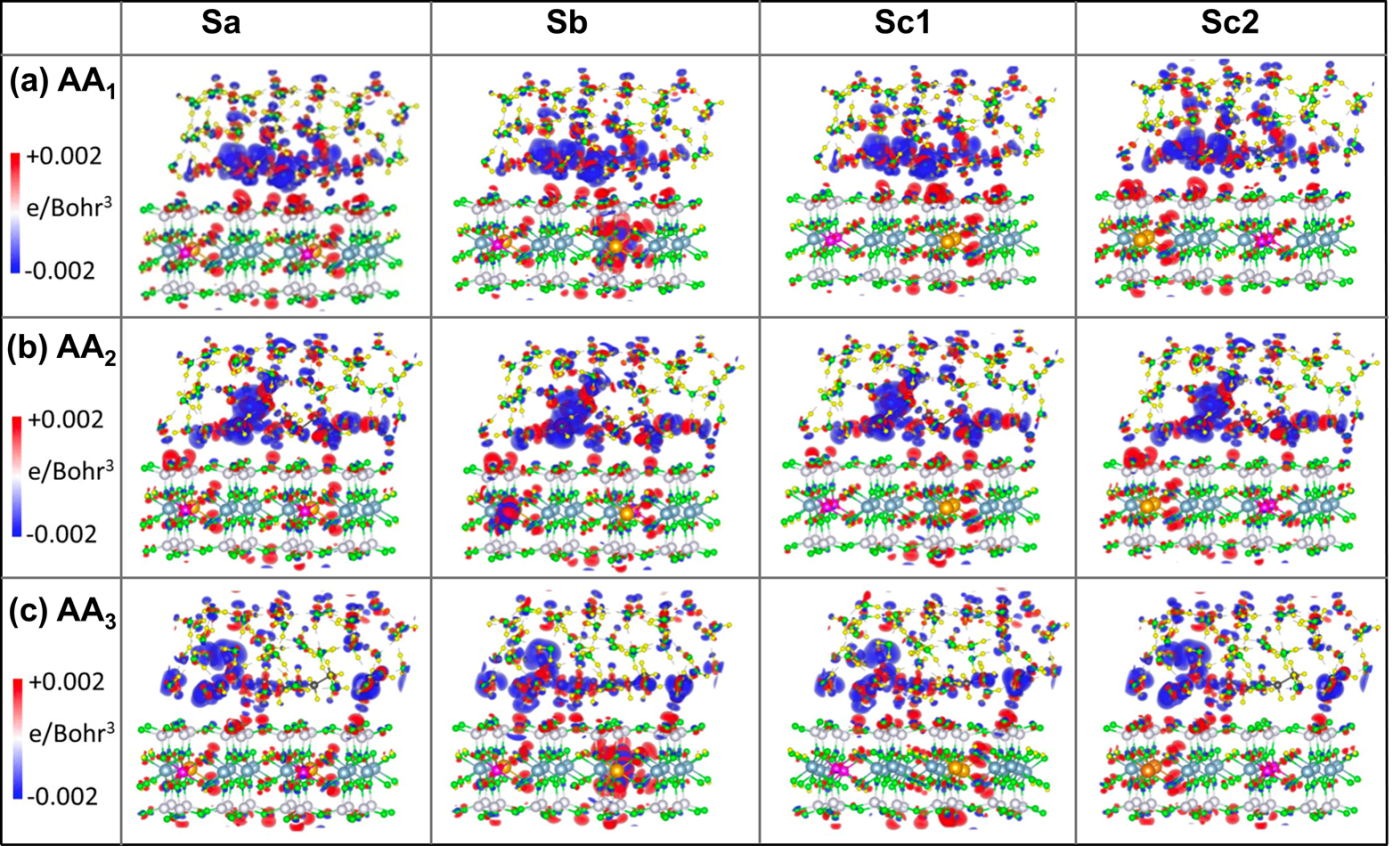
3D iso-surface plots of charge density difference (Δρ) caused by the adsorption of AA molecules (a) AA1, (b) AA2, and (c) AA3 on different nanoclay models Sa, Sb, Sc1, and Sc2 in the aqueous medium. The iso-surface value ranges from −0.002 e/Bohr^3^ to +0.002 e/Bohr^3^, with the negative values (blue) representing the regions of charge depletion and the positive values (red) to the charge accumulation.

The charge density difference (Δρ) is calculated to characterize the net charge transfer between the AA molecule and the interacting atoms of the MMT nanoclay using eq 6 in the vacuum and eq 7 in the aqueous medium, respectively.
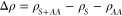
6
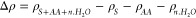
7The charge densities ρ_*S*_, ρ_*AA*_, and  are calculated based on the fixed geometry taken from the structures of interacting nanoclay (S), amino acid (AA), and the water (n.H_2_O) molecules. The 3D iso-surface plots in Figures 6 and 7 show charge density differences (Δρ) for all studied systems in the vacuum and in the water, respectively. Blue indicates charge depletion, while red shows charge accumulation due to the nanoclay and AA interaction.

The Δρ suggests that there is an exchange of electrons between the AA molecule and the nanoclay through the surface oxygens on the siloxane plane. The surface oxygens accumulate, and the AA molecule depletes the charge at the interacting sites both in the vacuum and in the water, Figures 6 and 7. This points to a strong electrostatic interaction between the AA and the nanoclay. The amine (−NH_2_) and carboxylic (−COOH) groups in the AA molecules contribute significantly to the adsorption, interacting strongly with the nearest oxygen species on the siloxane plane of the nanoclay. This is evidenced by the largest change in their Δρ. As expected, Δρ reflects the strong electron-donating character of the −NH_2_ group with a wider volume of electron depletion region than the electron-withdrawing group −COOH, even when −NH_2_ does not directly interact with the nanoclay surface (AA2 and AA3) (Figure 6).

These trends are supported by the Bader charge analysis^47,48^ representing the oxidation state (OS) of atom types in the nanoclay (Table S5) and interacting nanoclay with AA molecules (Table S6). When the AA molecules strongly interact with the slab, oxygen atoms in the −COOH group have an average OS that is more positive than those in the nanoclay oxygens by about 0.1, indicating charge donation by −COOH oxygens and charge acceptance by nanoclay oxygens. Additionally, the OS of nitrogen in the −NH_2_ group of interacting AA is about 0.3–0.5 more positive than its normal OS value of −3.0 in pristine AA molecules, suggesting an increase in electron donation when the AA interacts with the nanoclay slab.

Comparing the charge density difference plots in the vacuum (Figure 6) and in the water (Figure 7), we observe an increase in the number of physisorption sites at the nanoclay surface with a large Δρ in the presence of water molecules. This indicates that water enhances the binding affinity of the AA molecules for the nanoclay surface, which is consistent with the calculated nanoclay–AA interaction energies shown in Figure 4. Furthermore, water molecules show significant changes in their charge density due to interactions with both the AA molecule and the nanoclay, which confirms their strong contribution to the AA–nanoclay interaction. As expected, Δρ is larger for water molecules surrounding (−NH_2_) and −COOH groups, compared to those of −CH_2_ groups in the AA molecules, while the −CH_2_ groups also have negligible change in their charge density. This implies that the neutral −CH_2_ groups play an insignificant role in the interactions between the nanoclay and the AA molecules, both in the vacuum and in the water media.

Interestingly, while Mg(II) dopants remain unchanged in their charge density (Δρ = 0), most nanoclay models exhibit a slight accumulation of electron density around the Fe(II) dopants and their neighboring oxygens upon AA–nanoclay interactions. This electron accumulation (Δρ shown with a red color in Figures 6 and 7) varies in intensity among the nanoclays. The nanoclays that interact most strongly (Sc1 and Sc2) and have the smallest Fe–Fe separation show the least electron accumulation, regardless of the medium (vacuum or water). This observation is supported by the Bader charge analysis, which evidences a slight decrease in the average oxidation state of Fe from about +2.03 in the noninteracting slabs to +2.02 in the nanoclays strongly interacting with AA molecules, both in the vacuum and in the water (Tables S5 and S6). While small changes in Δρ indicate some electron transfer to Fe sites, occurring via hybridization with the closest oxygens both in the OH groups in the octahedral sheet and in the tetrahedral sheet of the nanoclay, the overall oxidation state demonstrates the stability of Fe(II) upon its interaction with the AA molecules, both in the vacuum and in the water.

In contrast, the Sb slab, which has the largest Fe–Fe separation and interacts least with the AA molecules in water, exhibits the largest Δρ for one of the Fe(II) atoms, but with noticeable electron depletion. In the vacuum, this trend is less consistent. The largest Δρ and electron depletion from Fe to the oxygens on the bottom siloxane plane are observed only for the Sb slab interacting with AA2, while this feature is seen for AA1 and AA3 interacting with the Sc1 nanoclay, which has the smallest Fe–Fe separation. This variability may be attributed to the stronger dipole moments of AA1 and AA3 molecules compared to AA2, which differently disturb the electron density of the nanoclay with the smallest Fe–Fe separation. Interactions with water molecules reduce the overall dipole moments of both AA molecules and the nanoclay surface. This results in fewer variations between interacting molecules, so that the change in charge density and electron depletion from Fe dopants are now governed only by their largest separation and weakest AA–nanoclay interactions. As such, weak interactions between the AA and the nanoclay demonstrate a propensity for the oxidation of Fe(II) to Fe(III).

It is well known that bulk MMT clay contains some portion of Fe(III) in the octahedral sites through the oxidation of Fe(II) to Fe(III).^42^ The reverse reaction of Fe(III) to Fe(II) reduction can be governed by different reducing agents, including dithionite and bacteria, despite the distant location of the octahedral Fe from the point of closest approach by any reducing agent.^43^ The calculated trends in electron donation and acceptance within the octahedral sheet of the nanoclay reveal the ability of AA molecules to stabilize Fe(II) and prevent its oxidation to Fe(III) due to strong AA–nanoclay interactions.

### Determination of the Magnetic Order

The replacement of octahedral Al(III) sites by Fe(II) and Mg(II) dopants introduces an excess negative charge into the system, leading to a net spin moment in the MMT nanoclays originating from the partially filled *d* orbital of Fe atoms.^41^ Consequently, the interactions between the AA and H_2_O molecules with the nanoclay surface are expected to impact the magnetic properties of these materials, which hold significance for their bioengineering applications. The magnesium atom, on the other hand, neither shows a spontaneous nor induced magnetic moment during the interaction with the AA molecule, both in the vacuum as well as in the aqueous medium, consistent with the study performed by Luna et al.^49^ on isomorphic substitution of Fe–Mg in delaminated pyrophyllite (D-P). However, the role of the Mg dopant relies on enhancing the cation exchange capacity, adsorption capabilities, and structural stability of such systems.^49−51^ To identify the magnetic order in the MMT nanoclay models, with and without adsorbed AA molecules, in both the vacuum and aqueous medium, we perform purely collinear DFT simulations. Incorporating vector-spin DFT, we assess the spin–spin interactions among the atoms of the nanoclay, AA, and water molecules. This involves calculating the total energy for various magnetic orders, including ferromagnetic (FM), antiferromagnetic (AFM), ferrimagnetic (FEM), and nonmagnetic (NM) states in the free nanoclay slab models without adsorbates and interacting nanoclays with the AA molecules, both in the vacuum and in the water. For each magnetic order, there exist two possible configurations, as illustrated in Figure S3. From these two configurations corresponding to the same magnetic order, the energetically favored configuration is chosen for comparison with the other magnetic orders. The exchange coupling between different magnetic orders is determined by comparing their energies to that of the nonmagnetic (*E*_*NM*_) system and represented as *E_M_– E_NM_*, where *M* stands for FM, AFM, or FEM. Thus, the ground state condition for the FM, AFM, or FEM state of the system is determined by the lowest energy configuration, as governed by the exchange coupling expressed in eq 8:
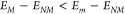
8where *M* and *m* represent FM, AFM, or FEM configurations and *M* ≠ *m*. If the condition given by eq 8 is valid, then the configuration *M* is more stable than *m*. After predicting the stable ground state magnetic ordering among FM, AFM, and FEM configurations of each MMT nanoclay model, the total magnetic moment *m*_tot_ (μ_*B*_) is estimated along with the magnitude of the local magnetic moment on two Fe(II) ions, and the magnetic contributions from the N and O atoms in the interacting AA molecules. These values are reported in Table 1.

**Table 1:** Total Magnetic Moment (*m*_tot_) Per Supercell (2 × 2 × 1), the Local Magnetic Moment on Each Fe(II) Dopant (*m*_Fe1_ and *m*_Fe2_) of the MMT Nanoclay and on the Nitrogen Atom (*m*_*N*_) and the Oxygen Atom () of the AA Molecule, and the Most Stable Magnetic Order Due to the Adsorption of Amino Acid Molecules AA1, AA2, and AA3 on the Nanoclay Model Sa, Sb, Sc1, and Sc2 in the Vacuum and in the Aqueous Medium (+51.H_2_O)^a^^b^

Slab	AA	*m*_tot_	*m*_Fe1_	*m_Fe2_*	*m*_*N*_	*m_O__–AA_*	magnetic order
		(μ_*B*_)	(μ_*B*_)	(μ_*B*_)	(μ_*B*_)	(μ_*B*_)	
Sa		+3.749	–2.121	+4.300	+0.360	+0.053	FEM
Sb	AA1	0.000	–4.291	+4.244	+0.371	+0.068	AFM/FM
Sc1		+3.999	+4.304	–2.376	+0.346	+0.072	FEM/FM
Sc2		+9.824	+4.301	+4.302	–0.014	+0.052	FM*
Sa		0.000	–4.306	+4.303	–0.504	+0.069	AFM
Sb	AA1 + 51.H_2_O	+10.080	+4.315	+4.303	+0.496	–0.065	FM
Sc1		+11.999	+4.304	+4.303	+0.504	+0.079	FM
Sc2		+10.003	+4.303	+4.305	+0.322	–0.061	FM*
Sa		+10.011	+4.268	+4.233	+0.218	+0.084	FM
Sb	AA2	0.000	–4.297	+4.217	+0.214	+0.081	AFM
Sc1		0.000	+4.304	–4.303	+0.210	+0.084	AFM*
Sc2		0.000	–4.304	+4.301	+0.221	+0.023	AFM
Sa		0.000	–4.305	+4.305	–0.343	+0.057	AFM/FM
Sb	AA2 + 51.H_2_O	+9.614	+4.304	+4.303	–0.321	+0.008	FM*
Sc1		+10.133	+4.304	+4.303	+0.397	–0.063	FM
Sc2		+9.917	+4.305	+4.304	–0.388	+0.019	FM
Sa		+11.343	+4.249	+4.275	+0.246	+0.082	FM
Sb	AA3	+9.963	+4.230	+4.278	+0.245	+0.084	FM
Sc1		0.000	+4.302	–4.304	+0.236	+0.075	AFM*
Sc2		+11.038	+4.301	+4.301	+0.250	+0.093	FM
Sa		0.000	–4.304	+4.302	–0.355	+0.048	AFM*/FM
Sb	AA3 + 51.H_2_O	+11.373	+4.311	+4.303	+0.277	+0.034	FM
Sc1		0.000	+4.304	–4.302	+0.333	–0.038	AFM
Sc2		+11.969	+4.304	+4.303	–0.358	+0.052	FM

a Here, the magnetic moment on the O-AA atom represents the average of all the oxygen atoms in the AA molecules. The slash denotes the magnetic order associated with an energy difference less than 6 meV.

b The asterisk denotes the most stable magnetic configuration, corresponding to the lowest energy of the interacting nanoclay model, compared to all others, interacting with the same AA molecule.

In noninteracting scenarios, the results indicate that all free nanoclay slab models Sa, Sb, and Sc exhibit FM behavior, as depicted in Figure S4. Among these configurations, the Sb nanoclay, characterized by the largest Fe–Fe separation, displays the strongest stability in the FM state, with its energy being 7 meV lower than that of Sc and 525 meV lower than that of Sa. However, the energy of the Sb slab in its FM state is only 1 meV lower than its AFM state, suggesting competition between FM and AFM states at temperatures exceeding 12 K. In the study performed by Fu and Yang on single Fe-doped Kaolinite clay, which is of similar chemical composition to that of MMT nanoclay, the ground state magnetic order is AFM.^13^ In our study, the Fe–Mg codoping further enhances the ion-exchange capacity with ferromagnetic stability. The calculated value of the total magnetic moment in the free nanoclay shows that the Sa and Sb nanoclay with the largest Fe–Fe separations have the largest magnetic moment of 9.758μ_*B*_ and 9.731μ_*B*_, respectively, while the nanoclay with the closest Fe–Fe separation has the smallest magnetic moment of 7.995μ_*B*_. These results are in agreement with experimental observations, which have reported the ferromagnetic stability and increased magnetization due to the incorporation of magnetic dopants in the MMT nanoclay.^52^ Other experiments have shown that unaltered iron-rich MMT nanoclays typically exhibit AFM order. However, in reduced samples containing only Fe(II), the exchange interaction shifts to a superparamagnetic or spin glass state at low temperatures, transitioning to an FM state as the temperature increases.^52^ Qualitatively, the calculations presented in this paper are consistent with these experimental findings.

**Figure 8:**
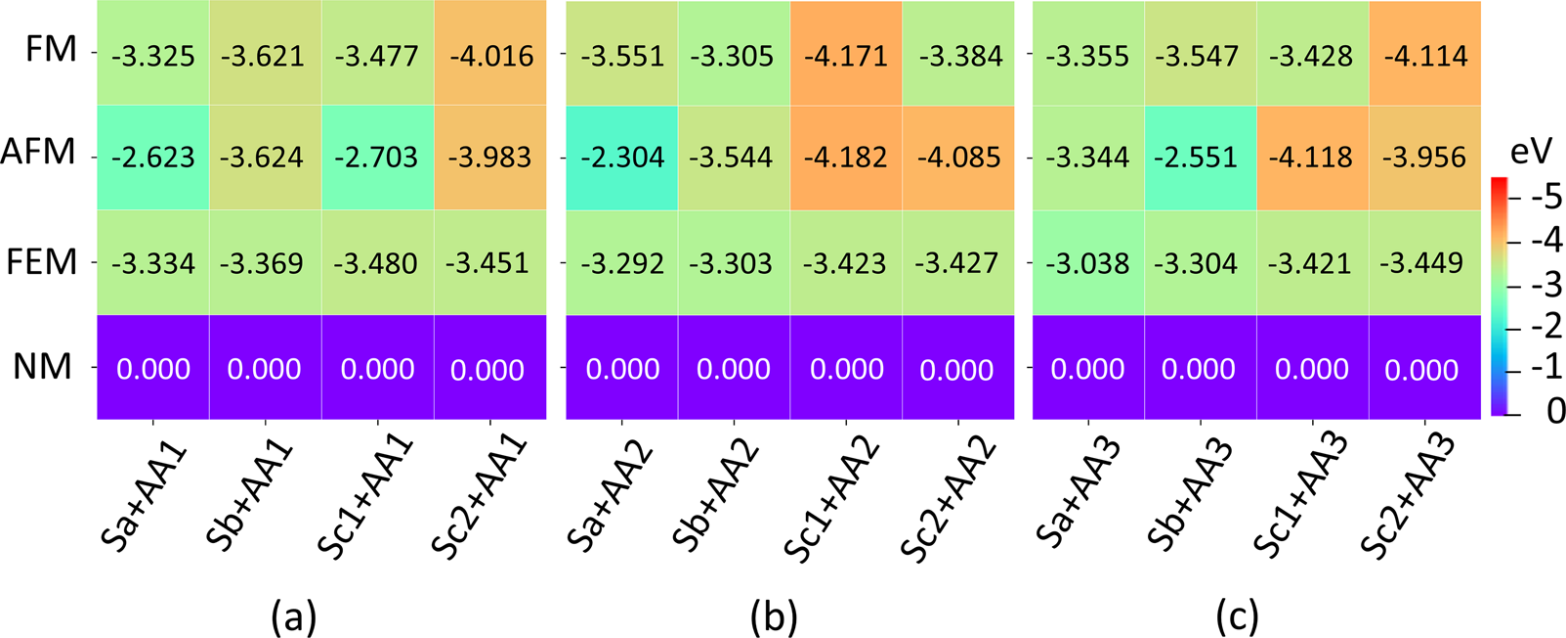
Heat map representing magnetic order competition in the nanoclay models Sa, Sb, Sc1, and Sc2 due to the adsorption of the amino acid molecules (a) AA1, (b) AA2, and (c) AA3 in the vacuum. Here, the numbers in the inset represent the relative energy values (in eV) of different magnetic orders, FM, AFM, and FEM, with respect to the nonmagnetic (NM) configuration, referenced to zero.

**Figure 9:**
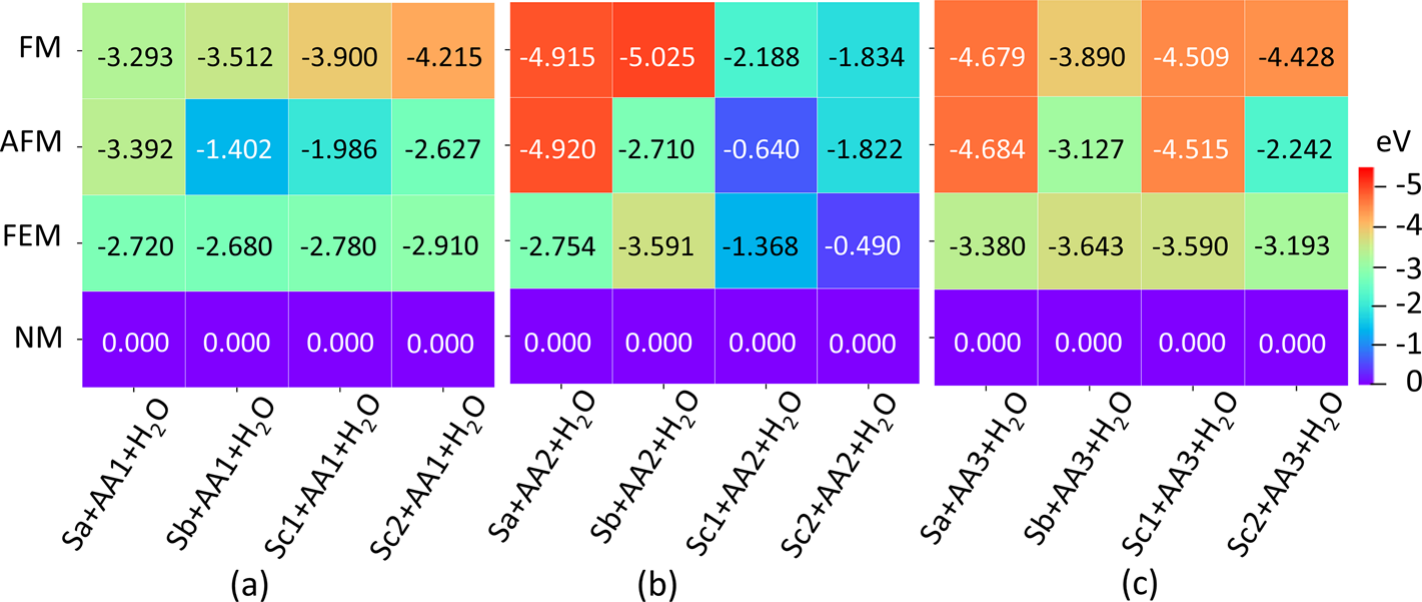
Heat map representing magnetic order competition in the nanoclay models Sa, Sb, Sc1, and Sc2 due to the adsorption of the amino acid molecules (a) AA1, (b) AA2, and (c) AA3 in the aqueous medium. Here, the numbers in the inset represent the relative energy values (in eV) of different magnetic orders, FM, AFM, and FEM, with respect to the nonmagnetic (NM) configuration, referenced to zero.

The interaction between the MMT nanoclay and the AA molecules stabilizes the AFM over FM order in some nanoclay configurations, as shown in the heating maps in Figures 8 and 9 for vacuum and water environments, respectively. This transition is highly sensitive to the particular AA molecule and its configuration on the slab. Thus, in the vacuum, the adsorption of the AA2 molecule leads to the AFM order in all slabs, except Sa (Figure 8). Similarly, the adsorption of AA3 to the nanoclay in the Sc1 configuration and AA1 in the Sb configuration also results in AFM order in the vacuum. However, the AA1 molecule interacting with the Sb nanoclay provides the energy of the AFM state only 3 meV lower compared to its FM state, suggesting a competition between AFM and FM order at temperatures higher than 35 K. In an aqueous medium, the Sa nanoclay tends to exhibit a more consistent preference for the AFM order, regardless of the AA type, as depicted in Figure 9. However, at temperatures above 60 K (5 meV), both AA2 and AA3 adsorbed on the Sa nanoclay in the presence of water molecules show a rivalry between AFM and FM order. Similarly, for AA3 in the Sc1 configuration in water, AFM is favored over FM, with an energy difference of 6 meV suggesting competition at temperatures higher than 70 K.

Based on the most stable configurations with the lowest energy, as identified by the orange-red colors in the heating maps in Figures 8 and 9, the trends are outlined as to how the AA–nanoclay interactions impact transitions between FM and AFM order. In both vacuum and aqueous environments, the interaction with the AA1 molecule strongly stabilizes the FM state in the Sc2 nanoclay configuration, characterized by the shortest Fe–Fe separation. While the Sc2 configuration remains the most stable when interacting with AA2 and AA3 in the vacuum, these interactions tend to favor the AFM state, albeit with energy lower than that of the FM order by only 11 meV and 4 meV, respectively. Conversely, in aqueous media, AA2 and AA3 stabilize the slabs with larger Fe–Fe separation (Sa and Sb). However, AA2 strongly favors FM over AFM when interacting with the Sb slab, while AA3 interacting with the Sa nanoclay leans toward stabilizing the AFM over FM order, with a small competing energy of 5 meV between these states. These distinct trends can be rationalized by the variations in the AA structures: AA1 interacts with the nanoclay via the amine and carboxyl groups, AA2 interacts via the two carboxyl groups, and AA3 interacts via the one carboxyl group.

**Figure 10:**
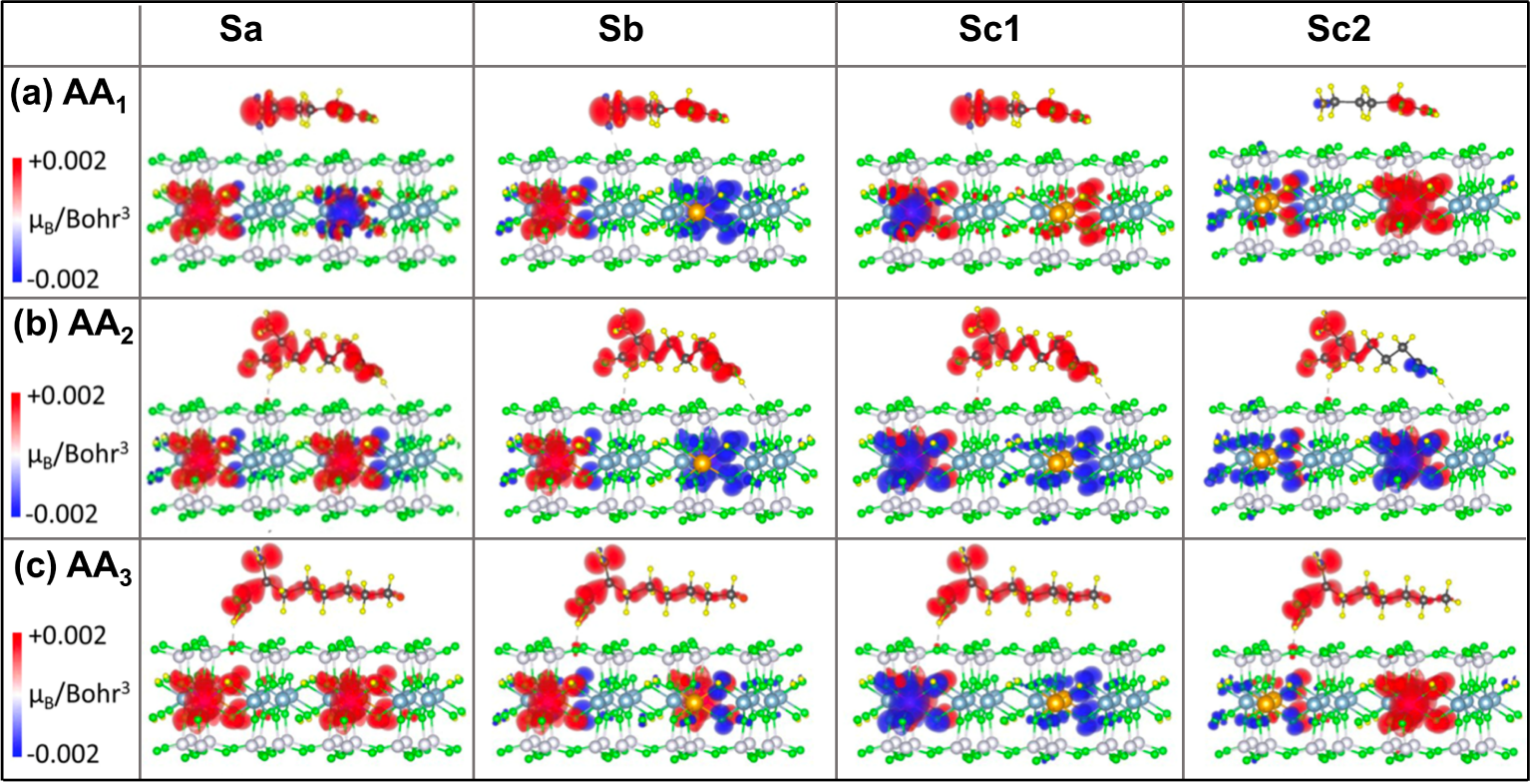
Diagrams representing the 3D iso-surface plots showing the magnetization density map (MDM) for the adsorbed AA molecules (a) AA1, (b) AA2, and (c) AA3 on different nanoclay models Sa, Sb, Sc1, and Sc2 in the vacuum. To discern the value of magnetic moment qualitatively, the iso-surface range of −0.002 *μ*_*B*_/Bohr^3^ (blue) to +0.002 *μ*_*B*_/Bohr^3^ (red) is chosen.

**Figure 11:**
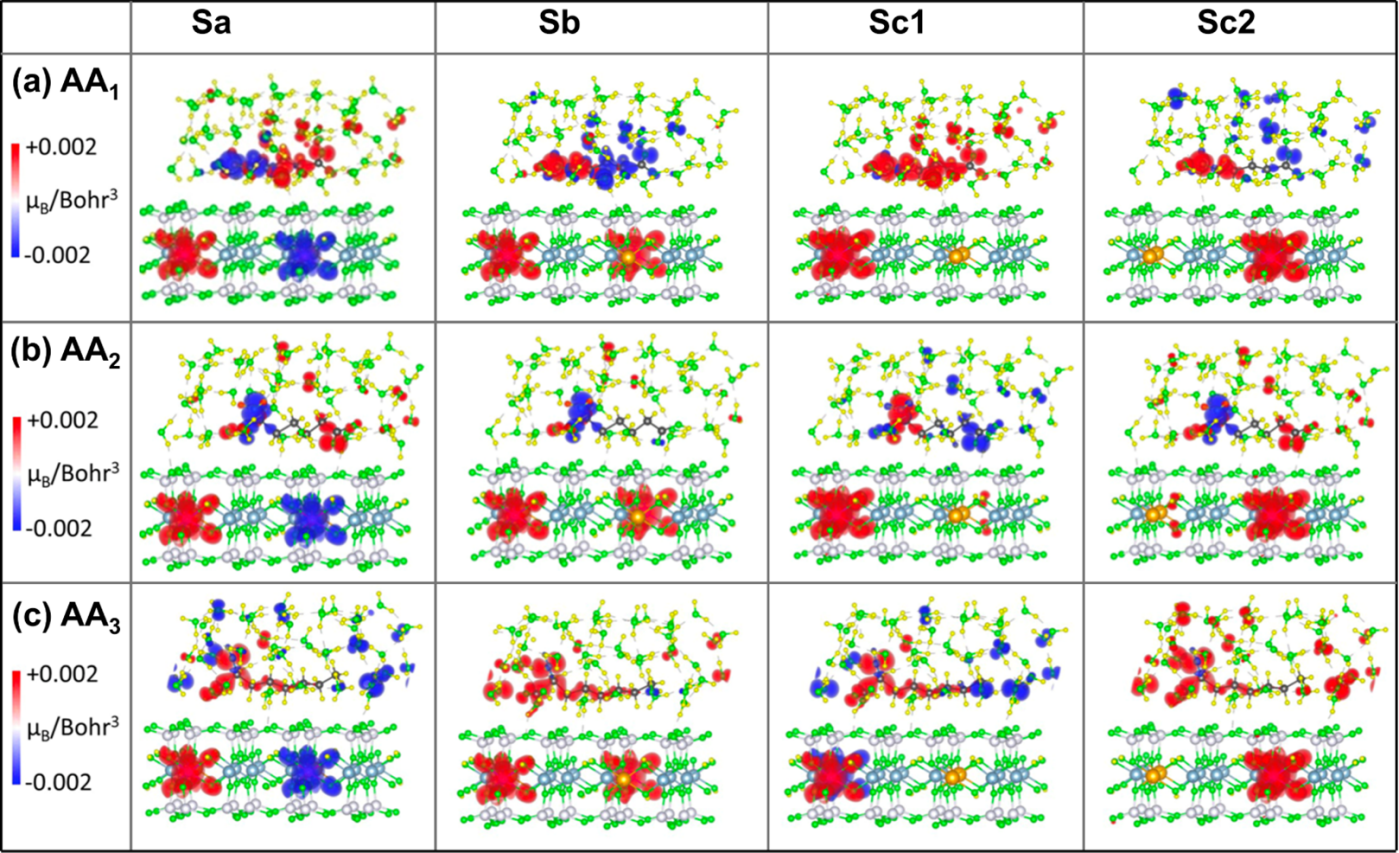
Diagrams representing the 3D iso-surface plots of the magnetization density map (MDM) of the adsorbed AA molecules (a) AA1, (b) AA2, and (c) AA3 on different nanoclay models Sa, Sb, Sc1, and Sc2 in the aqueous medium. To discern the value of magnetic moment qualitatively, the iso-surface range of −0.002 *μ*_*B*_/Bohr^3^ (blue) to +0.002 *μ*_*B*_/Bohr^3^ (red) is chosen.

To gain more insights into the correlations between the interacting AA molecules and the magnetic properties of the nanoclay, additional information regarding magnetization density is extracted using the difference between spin-up and spin-down components, *ρ*^↑^ – *ρ*^↓^, from the total charge density, where *ρ*^↑^ and *ρ*^↓^ represents the charge density distribution along the spin-up and spin-down directions, respectively.^53^ The 3D iso-surface plots of the magnetization density maps (MDMs) show the spontaneous magnetic moment produced on the Fe(II) ions and the inductive magnetic moment on the AA molecule in vacuum, Figure 10. The effect of an aqueous solution on this magnetic moment is depicted in Figure 11. In Figures 10 and 11, the red and blue colors in the MDM diagram represent the magnetic moment in the spin-up and spin-down directions, respectively.

In the vacuum, all interacting AA molecules exhibit induced magnetic moments, with spin-up mainly localized along each molecule’s chain, regardless of the nanoclay configuration and its magnetic order, as illustrated in Figure 10. However, there is an exception with the Sc2 nanoclay interacting with the AA1 and AA2 molecules, where the spin density includes both up and down spin components, each localized on different parts of the molecules. This difference is attributed to the competition between the interacting amine and carboxyl groups in AA1 and the two carboxyl groups in AA2. This competition arises when the carboxyl group is positioned closest to the nanoclay surface (Tables S1 and S2) and oriented toward the least separated Fe atoms.

It is worth noting that the interplay between the electron-donating amine group and the electron-withdrawing carboxyl group in AA1, engaged in interactions with the nanoclay, results in the stabilization of FEM states for the Sa and Sc1 nanoclay configurations in the vacuum. These configurations are characterized by the amine group being positioned close to the nanoclay surface (Table S1) and oriented toward an iron, as demonstrated in Figure 10a. In the FEM state, the spin alignment resembles that of the AFM state with opposite spin directions on each iron. However, the total magnetic moment of the FEM state falls between those of the AFM (zero value) and FM (maximum value) states. This difference stems from the unequal magnitudes of magnetic moments between the two Fe atoms, as shown in Table 1, which occurs due to the donation of some charge density from one Fe to the nearest oxygens. FEM materials have promising properties for spintronic applications, including adjustable magnetization and ultrafast magnetic dynamics via changes in chemical composition and temperature.^54^

In an aqueous environment, however, the FEM order is energetically unfavorable for all nanoclay configurations interacting with AA1, as well as with other AA molecules, as shown in the heat map in Figure 9. Thus, the redistribution of charge densities over water molecules balances the magnitudes of the magnetic moments between the two Fe atoms in the nanoclays. Additionally, the presence of water tends to reduce the spin polarization of the AA molecules, resulting in both up and down spin components localized on different parts of the AA molecules interacting with various nanoclay configurations, independent of their magnetic order, as depicted in Figure 11. However, there are exceptions, such as AA3 interacting with all nanoclay configurations and AA1 interacting with the Sc1 nanoclay. In these cases, the spin density of the AA molecule remains polarized, with a dominant spin-up contribution, although not as strongly as in the vacuum. The reduced spin polarization of the AA molecules is attributed to the magnetic moment induced on the nearest water molecules, as illustrated in Figure 11 and quantitatively presented in Table 1. The additional effect of water on the nanoclays with adsorbed AA molecules is to enhance the total magnetic moment of the nanoclay configurations exhibiting the FM order, as indicated in Table 1. This increase in magnetic moment is more pronounced for interacting AA3 and AA1, displaying a highly induced spin polarization, and enhancing the degree of ferromagnetism.

Finally, the magnetic properties have been compared using U_eff_ = 3.0 eV and 4.0 eV within the same supercell size of 2×2×1 of the nanoclay model (Sa), for the adsorption of AA1 and AA2 molecules in the vacuum. The total magnetic moment (m_tot_) per supercell, the local magnetic moment on each Fe (II) dopant (m_Fe1_ and m_Fe2_), and the most stable ground state magnetic order are shown in Table.S7. Here, the ground state magnetic order remains consistent for the U_eff_ values. However, the local magnetic moment on Fe (II) atoms is more stabilized in U_eff_ = 4.0 eV compared to U_eff_ = 3.0 eV. This indicates that our choice of U_eff_ = 4.0 eV throughout the paper is more accurate and valid.

### Signature of a Quantum Ferrofluid

The calculations show that out of 12 interacting nanoclay configurations (4 configurations with each AA molecule), 8 configurations lead to FM stability in an aqueous medium, such as (Sb, Sc1, Sc2) + AA1, (Sb, Sc1, Sc2) + AA2, and (Sb, Sc2) + AA3, as shown in Table 1. The enhanced ferromagnetism in most of the nanoclays interacting with the AA molecules in water is rationalized by the magnetization of some closely located H_2_O molecules, resulting from the coupled magnetic interaction between Fe(II) dopants in the nanoclay and the AA molecules adsorbed on the nanoclay surface. The magnetism induced in the H_2_O molecules may exhibit parallel or antiparallel orientations concerning the FM or AFM coupling with Fe(II) dopants in the nanoclay, as illustrated in Figure 11. However, majority of the spin-polarized H_2_O molecules in these nanoclay configurations interact with each other ferromagnetically, exhibiting either spin up or spin down orientations, thereby behaving as a single quantum mechanical entity.

The predicted ferromagnetic stability in an aqueous medium in these nanoclay models interacting with the AA molecules suggests the emergence of magnetic colloids or aqueous ferrofluids observed in similar systems.^55−57^ Ferrofluid refers to a system where the magnetic nanoparticles are suspended in the liquid, carrying the properties of both the liquid and the magnet.^58^ Ferrofluid has several exciting biomedical and optical applications. They are extensively utilized in preparing optical switches and biosensors, treating hyperthermia and tumor cells, operating magnetic resonance imaging (MRI)-based drug delivery, quantifying biomolecule agglutination, etc.^59^ Further, the magnetic nature of water molecules may cause an unusual hydrodynamic effect. Thus, an increase in magnetization in an aqueous medium compared to vacuum systems is known to cause a Rosensweig instability, creating spin self-ordered regions.^60^ This process can benefit from the predicted increment in the binding affinity of the AA molecules in the water medium compared to vacuum.

## Conclusions

The performed spin-polarized DFT-based computations provide atomistic insights into the electrostatic and magnetic properties, along with their dependence on interactions between the MMT nanoclay and adsorbed AA molecules. The calculated interaction or binding energies (*E_b_*) between AA molecules and the MMT nanoclay demonstrate a strong binding affinity, with the absolute values ranging from 1.5 to 2.3 eV, depending on the AA type and its alignment on the nanoclay. The binding affinity increases 2–3 times in the presence of water molecules. The nanoclay with the shortest Fe–Fe separation (Sc1 and Sc2) most strongly interacts with the AA molecules, compared to nanoclay with a longer Fe–Fe separation (Sa and Sb), both in the vacuum and in the aqueous medium. The calculated charge density difference (Δρ) indicates that the AA–nanoclay interaction is facilitated through electron donation from the AA to the nanoclay. This process is largely contributed by the surface oxygen atoms at the adsorption sites in the siloxane plane of the nanoclay and the amine and carboxylic groups of the AA molecule. This charge accumulation on the surface oxygen atoms is significantly dispersed toward more oxygen atoms in the aqueous medium. We also observed a strong variations in the charge density on the dopant Fe atoms in some of the nanoclay models, as well as on their nearest oxygen atoms in the octahedral sheet. The calculations demonstrate that the AA molecules have the ability to stabilize Fe(II) and prevent its oxidation to Fe(III) via strong electrostatic interactions with the nanoclay.

The variation in charge density in the vicinity of dopant atoms is found to be accompanied by significant changes in the magnetic dipole moments of the systems. This leads to a tunable magnetic order of FM, FEM, or AFM, depending on the type of AA molecule and its alignment on the nanoclay surface. Additionally, our calculations unveil a significant finding: Prevailing magnetic exchange interactions are observed, driven by spin transfer from the Fe(II) dopants within the interior of the MMT nanoclay to the AA molecule, and extending to a long-range in the interacting water molecules. Our results show the spin-polarization of water molecules induced by the nanoclay–AA interactions, causing them to interact with each other ferromagnetically, thus behaving as a unified quantum mechanical entity. Moreover, the ferromagnetism in most of the MMT–AA configurations has been enhanced in the presence of an aqueous medium, indicating the signature of permanent aqueous-ferrofluid in these systems. The water nanoparticles exhibiting magnetic coupling with Fe(II) ions of the MMT nanoclay clearly describe the phenomenon of ferrohydrodynamics potentially leading to Rosensweig instability. This study serves as an archetype of novel research on these surface interactions. To fully understand the long-range spin polarization and ferrofluid behavior observed, future research should apply nonadiabatic dynamics.^61^ Our theoretical predictions on the stabilization of Fe(II) and the enhancement of ferromagnetism in aqueous media could open new avenues in the discovery of next-generation quantum ferrofluid with a plethora of applications in biomedical, optical, and electrochemical fields.

## Supporting Information Available

Relaxed primitive geometrical structures of pristine bulk MMT clay and a single layer nanoclay of MMT; diagram representing alignment of the three different amino acids AA1, AA2, and AA3, on the nanoclay model Sa; calculated value of the AA-nanoclay binding energy (*E*_*b*_) in eV; minimum adsorption height (*h*_min_) and the height of different atoms on AA1, AA2, and AA3 molecules; the charge transfer (oxidation states) of different atoms in nanoclay models Sa, Sb, and Sc, before and after the adsorption; diagram illustrating different magnetic orders; heat map representing magnetic order competition in three different free slab models before the adsorption of amino acids; and the comparison of total and local magnetic moments using *U*_eff_ = 3.0 eV and *U*_eff_ = 4.0 eV during the adsorption of AA1 molecule on the nanoclay model Sa in the vacuum medium (PDF)

## References

[ref1] Tran A. T.; Patterson D. A.; James B. J. Investigating the feasibility of using polysulfone–montmorillonite composite membranes for protein adsorption. J. Food Eng. 2012, 112, 38–49. 10.1016/j.jfoodeng.2012.03.031.

[ref2] Corrales T.; Larraza I.; Catalina F.; Portolés T.; Ramírez-Santillán C.; Matesanz M.; Abrusci C. In vitro biocompatibility and antimicrobial activity of poly(ε-caprolactone)/montmorillonite nanocomposites. Biomacromolecules 2012, 13, 4247–4256. 10.1021/bm301537g. 23153018

[ref3] Ma Y.-L.; Xu Z.-R.; Guo T.; You P. Adsorption of methylene blue on Cu (II)-exchanged montmorillonite. J. Colloid Interface Sci. 2004, 280, 283–288. 10.1016/j.jcis.2004.08.044. 15533398

[ref4] Katti D. R.; Srinivasamurthy L.; Katti K. S. Molecular modeling of initiation of interlayer swelling in Na–montmorillonite expansive clay. Can. Geotech. J. 2015, 52, 1385–1395. 10.1139/cgj-2014-0309.

[ref5] Cai P.; Huang Q.; Li M.; Liang W. Binding and degradation of DNA on montmorillonite coated by hydroxyl aluminum species. Colloids Surf., B 2008, 62, 299–306. 10.1016/j.colsurfb.2007.10.016. 18055187

[ref6] Mignon P.; Sodupe M. Theoretical study of the adsorption of DNA bases on the acidic external surface of montmorillonite. Phys. Chem. Chem. Phys. 2012, 14, 945–954. 10.1039/C1CP22454A. 22124483

[ref7] Yu W. H.; Li N.; Tong D. S.; Zhou C. H.; Lin C. X. C.; Xu C. Y. Adsorption of proteins and nucleic acids on clay minerals and their interactions: A review. Appl. Clay Sci. 2013, 80, 443–452. 10.1016/j.clay.2013.06.003.

[ref8] Zhou X.; Huang Q.; Chen S.; Yu Z. Adsorption of the insecticidal protein of Bacillus thuringiensis on montmorillonite, kaolinite, silica, goethite and Red soil. Appl. Clay Sci. 2005, 30, 87–93. 10.1016/j.clay.2005.04.003.

[ref9] Fiorito T. M.; Icoz I.; Stotzky G. Adsorption and binding of the transgenic plant proteins, human serum albumin, β-glucuronidase, and Cry3Bb1, on montmorillonite and kaolinite: Microbial utilization and enzymatic activity of free and clay-bound proteins. Appl. Clay Sci. 2008, 39, 142–150. 10.1016/j.clay.2007.07.006.

[ref10] Ambre A.; Katti K. S.; Katti D. R. In situ mineralized hydroxyapatite on amino acid modified nanoclays as novel bone biomaterials. Mater. Sci. Eng. 2011, 31, 1017–1029. 10.1016/j.msec.2011.03.001. 25491979

[ref11] Katti K. S.; Ambre A. H.; Peterka N.; Katti D. R. Use of unnatural amino acids for design of novel organomodified clays as components of nanocomposite biomaterials. Philos. Trans. R. Soc., A: Math., Phys. Eng. Sci. 2010, 368, 1963–1980. 10.1098/rsta.2010.0008. 20308111

[ref12] Sainz-Díaz C. I.; Palin E. J.; Dove M. T.; Hernández-Laguna A. Monte Carlo simulations of ordering of Al, Fe, and Mg cations in the octahedral sheet of smectites and illites. Am. Mineral. 2003, 88, 1033–1045. 10.2138/am-2003-0712.

[ref13] Fu L.; Yang H. Structure and electronic properties of transition metal doped kaolinite nanoclay. Nanoscale Res. Lett. 2017, 12, 411 10.1186/s11671-017-2188-4. 28618720 PMC5471148

[ref14] Yun S. J.; Cho B. W.; Dinesh T.; Yang D. H.; Kim Y. I.; Jin J. W.; Yang S.-H.; Nguyen T. D.; Kim Y.-M.; Kim K. K.; et al. Escalating Ferromagnetic Order via Se-Vacancies Near Vanadium in WSe2Monolayers. Adv. Mater. 2022, 34, 2106551 10.1002/adma.202106551. 34962658

[ref15] Jiang D.; Wang J.; Gao R.; Fu L.; Yang H. Contrasting photochemical activity of two sub-layers for natural 2D nanoclay with an asymmetric layer structure. ACS Appl. Mater. Interfaces 2021, 13, 59431–59439. 10.1021/acsami.1c15029. 34855349

[ref16] Wang Y.; Jin X.; Peng A.; Gu C. Transformation and toxicity of environmental contaminants as influenced by Fe containing clay minerals: a review. Bull. Environ. Contam. Toxicol. 2020, 104, 8–14. 10.1007/s00128-019-02747-2. 31740979

[ref17] Ray S. S.; Okamoto M. Polymer/layered silicate nanocomposites: A review from preparation to processing. Prog. Polym. Sci. 2003, 28, 1539–1641. 10.1016/j.progpolymsci.2003.08.002.

[ref18] Alves J. L.; eRosa P. D.; Morales A. R. Evaluation of organic modification of montmorillonite with ionic and nonionic surfactants. Appl. Clay Sci. 2017, 150, 23–33. 10.1016/j.clay.2017.09.001.

[ref19] Blöchl P. E. Projector augmented-wave method. Phys. Rev. B 1994, 50, 17953 10.1103/PhysRevB.50.17953. 9976227

[ref20] Kresse G.; Furthmuller J. Efficient Iterative Schemes for ab Initio Total-Energy Calculations Using a Plane-Wave Basis Set. Phys. Rev. B 1996, 54, 11169–11186. 10.1103/PhysRevB.54.11169. 9984901

[ref21] Perdew J. P.; Chevary J. A.; Vosko S. H.; Jackson K. A.; Pederson M. R.; Singh D. J.; Fiolhais C. Atoms, molecules, solids, and surfaces: Applications of the generalized gradient approximation for exchange and correlation. Phys. Rev. B 1992, 46, 6671 10.1103/PhysRevB.46.6671. 10002368

[ref22] Perdew J. P.; Burke K.; Ernzerhof M. Generalized gradient approximation made simple. Phys. Rev. Lett. 1996, 77, 3865 10.1103/PhysRevLett.77.3865. 10062328

[ref23] Ghazanfari S.; Han Y.; Xia W.; Kilin D. S. First-Principles Study on Optoelectronic Properties of Fe-Doped Montmorillonite Clay. J. Phys. Chem. Lett. 2022, 13, 4257–4262. 10.1021/acs.jpclett.2c00697. 35522138

[ref24] Iyemperumal S. K.; Deskins N. A. Evaluating solvent effects at the aqueous/Pt (111) interface. ChemPhyschem 2017, 18, 2171–2190. 10.1002/cphc.201700162. 28464413

[ref25] Steinmann S. N.; Sautet P.; Michel C. Solvation free energies for periodic surfaces: comparison of implicit and explicit solvation models. Phys. Chem. Chem. Phys. 2016, 18, 31850–31861. 10.1039/C6CP04094B. 27841404

[ref26] Klimes J.; Bowler D. R.; Michaelides A. Van der Waals density functionals applied to solids. Phys. Rev. B 2011, 83, 195131 10.1103/PhysRevB.83.195131.

[ref27] Chiter F.; Nguyen V. B.; Tarrat N.; Benoit M.; Tang H.; Lacaze-Dufaure C. Effect of van der Waals corrections on DFT-computed metallic surface properties. Mater. Res. Express 2016, 3, 046501 10.1088/2053-1591/3/4/046501.

[ref28] Song H. Y.; Yoo B. I.; Choi J.-H.; Kang S.-H.; Bang J.; Li W.; Nandadasa C. N.; Thapa D.; Yoon D.; Han M. J.; Lee K. H.; Kim S. G.; Lee K.; Kim S. W. Van der Waals electride: Toward intrinsic two-dimensional ferromagnetism of spin-polarized anionic electrons. Mater. Today Phys. 2021, 20, 100473 10.1016/j.mtphys.2021.100473.

[ref29] Momma K.; Izumi F. VESTA 3 for three-dimensional visualization of crystal, volumetric and morphology data. J. Appl. Crystallogr. 2011, 44, 1272–1276. 10.1107/S0021889811038970.

[ref30] Horton M. K.; Montoya J. H.; Liu M.; Persson K. A. High-throughput prediction of the ground-state collinear magnetic order of inorganic materials using density functional theory. Npj Comput. Mater. 2019, 5, 64 10.1038/s41524-019-0199-7.

[ref31] Dudarev S.; Botton G.; Savrasov S.; Humphreys C.; Sutton A. Electron-energy-loss spectra and the structural stability of nickel oxide: An LSDA+ U study. Phys. Rev. B 1998, 57, 1505 10.1103/PhysRevB.57.1505.

[ref32] Wang L.; Maxisch T.; Ceder G. Oxidation energies of transition metal oxides within the GGA+ U framework. Phys. Rev. B 2006, 73, 195107 10.1103/PhysRevB.73.195107.

[ref33] Tolba S. A.; Gameel K. M.; Ali B. A.; Almossalami H. A.; Allam N. K. The DFT+ U: Approaches, accuracy, and applications. In Density Functional Calculations - Recent Progresses of Theory and Application; IntechOpen Limited, 2018; pp. 3–30. DOI: 10.5772/intechopen.72020.

[ref34] Neaton J.; Ederer C.; Waghmare U.; Spaldin N.; Rabe K. First-principles study of spontaneous polarization in multiferroic Bi Fe O 3. Phys. Rev. B 2005, 71, 014113 10.1103/PhysRevB.71.014113.

[ref35] Dixit V.; Thapa D.; Lamichhane B.; Nandadasa C. N.; Hong Y.-K.; Kim S.-G. Site preference and magnetic properties of Zn-Sn-substituted strontium hexaferrite. J. Appl. Phys. 2019, 125, 173901 10.1063/1.5084762.

[ref36] Fernando A.; Khan D.; Hoffmann M. R.; Çakır D. Exploring the biointerfaces: ab initio investigation of nano-montmorillonite clay, and its interaction with unnatural amino acids. Phys. Chem. Chem. Phys. 2023, 25, 29624–29632. 10.1039/D3CP02944A. 37881012

[ref37] Panchmatia P. M.; Sanyal B.; Oppeneer P. M. GGA+ U modeling of structural, electronic, and magnetic properties of iron porphyrin-type molecules. Chem. Phys. 2008, 343, 47–60. 10.1016/j.chemphys.2007.10.030.

[ref38] Scherlis D. A.; Cococcioni M.; Sit P.; Marzari N. Simulation of heme using DFT+ U: a step toward accurate spin-state energetics. J. Phys. Chem. B 2007, 111 (25), 7384–7391. 10.1021/jp070549l. 17547444

[ref39] Ferreira C. R.; Pulcinelli S. H.; Scolfaro L.; Borges P. D. Structural and electronic properties of iron-doped sodium montmorillonite clays: A first-principles DFT study. ACS Omega 2019, 4, 14369–14377. 10.1021/acsomega.9b00685. 31528789 PMC6740045

[ref40] Uddin F., Montmorillonite: an introduction to properties and utilization; IntechOpen: London, 2018.

[ref41] Sokolovskiy P.; Roessner F.; Vezentsev A.; Konkova T.; Alekhina M.; Manokhin S.; Greish A. Changes in the crystal lattice parameters of montmorillonite during its modification by cobalt and aluminum cations. Russ. J. Phys. Chem. A 2018, 92, 1947–1952. 10.1134/S0036024418100333.

[ref42] Lear P. R.; Stucki J. W. Magnetic properties and site occupancy of iron in nontronite. Clay Miner. 1990, 25, 3–13. 10.1180/claymin.1990.025.1.02.

[ref43] Stucki J. Properties and behaviour of iron in clay minerals. Dev. Clay Sci. 2006, 1, 423–475. 10.1016/S1572-4352(05)01013-5.

[ref44] Tian X.; Wang T.; Fan L.; Wang Y.; Lu H.; Mu Y. A DFT based method for calculating the surface energies of asymmetric MoP facets. Appl. Surf. Sci. 2018, 427, 357–362. 10.1016/j.apsusc.2017.08.172.

[ref45] Douillard J.; Salles F.; Devautour-Vinot S.; Manteghetti A.; Henry M. Study of the surface energy of montmorillonite using PACHA formalism. J. Colloid Interface Sci. 2007, 306, 175–182. 10.1016/j.jcis.2006.09.008. 17107683

[ref46] Gu C.; Liu C.; Johnston C. T.; Teppen B. J.; Li H.; Boyd S. A. Pentachlorophenol radical cations generated on Fe (III)-montmorillonite initiate octachlorodibenzo-p-dioxin formation in clays: density functional theory and fourier transform infrared studies. Environ. Sci. Technol. 2011, 45, 1399–1406. 10.1021/es103324z. 21254769 PMC3094738

[ref47] Henkelman G.; Arnaldsson A.; Jónsson H. A fast and robust algorithm for Bader decomposition of charge density. Comput. Mater. Sci. 2006, 36, 354–360. 10.1016/j.commatsci.2005.04.010.

[ref48] Benson D.; Sankey O. F.; Häussermann U. Electronic structure and chemical bonding of the electron-poor II-V semiconductors ZnSb and ZnAs. Phys. Rev. B 2011, 84, 125211 10.1103/PhysRevB.84.125211.

[ref49] Luna C. R.; Reimers W. G.; Avena M. J.; Juan A. Theoretical study of the octahedral substitution effect in delaminated pyrophyllite: physicochemical properties and applications. Phys. Chem. Chem. Phys. 2021, 23, 14601–14607. 10.1039/D1CP01032H. 34190255

[ref50] Bergaya F.; Lagaly G. Handbook of clay science; Newnes, 2013.

[ref51] Emmerich K.; Wolters F.; Kahr G.; Lagaly G. Clay profiling: the classification of montmorillonites. Clays Clay Miner. 2009, 57, 104–114. 10.1346/CCMN.2009.0570110.

[ref52] Jiraskova Y.; Bursik J.; Seidlerova J.; Kutlakova K. M.; Safarik I.; Safarikova M.; Pospiskova K.; Zivotsky O. Microstructural analysis and magnetic characterization of native and magnetically modified montmorillonite and vermiculite. J. Nanomater. 2018, 2018, 1–14. 10.1155/2018/3738106.

[ref53] Lee S. Y.; Bang J.; Song H. Y.; Il Yoo B.; Kim Y.; Lee K.; Thapa D.; Kim S.-G.; Kim S. W. Mixed-cation driven magnetic interaction of interstitial electrons for ferrimagnetic two-dimensional electride. NPJ. Quantum Mater. 2021, 6 (1), 21 10.1038/s41535-021-00319-4.

[ref54] Zhang Y.; Feng X.; Zheng Z.; Zhang Z.; Lin K.; Sun X.; Wang G.; Wang J.; Wei J.; Vallobra P.; et al. Ferrimagnets for spintronic devices: From materials to applications. Appl. Phys. Rev. 2023, 10 (1), 011301 10.1063/5.0104618.

[ref55] Berger P.; Adelman N. B.; Beckman K. J.; Campbell D. J.; Ellis A. B.; Lisensky G. C. Preparation and properties of an aqueous ferrofluid. J. Chem. Educ. 1999, 76, 943 10.1021/ed076p943.

[ref56] Scherer C.; Figueiredo Neto A. M. Ferrofluids: properties and applications. Braz. J. Phys. 2005, 35, 718–727. 10.1590/S0103-97332005000400018.

[ref57] Oldenburg C. M.; Borglin S. E.; Moridis G. J. Numerical simulation of ferrofluid flow for subsurface environmental engineering applications. Transp. Porous Media 2000, 38, 319–344. 10.1023/A:1006611702281.

[ref58] Verberck B. Made to order. Nat. Phys. 2016, 12, 205–205. 10.1038/nphys3688.

[ref59] Philip J.; Laskar J. M. Optical properties and applications of ferrofluids–a review. J. Nanofluids 2012, 1, 3–20. 10.1166/jon.2012.1002.

[ref60] Li Y.-H.; Huang K.-L. Effect of Rosensweig instability in a ferrofluid layer on reflection loss of a high-frequency electromagnetic wave. AIP Adv. 2022, 12 (4), 045221 10.1063/5.0086107.

[ref61] Kilina S.; Kilin D.; Tretiak S. Light-driven and phonon-assisted dynamics in organic and semiconductor nanostructures. Chem. Rev. 2015, 115, 5929–5978. 10.1021/acs.chemrev.5b00012. 25993511

