# Supplementary material for: Tunable Magnetic Order in Fe-Mg Codoped Montmorillonite Nanoclay Interfaced with Amino Acids
Source: ACS Omega. 2025 Jan 10;10(3):2539–52. doi: 10.1021/acsomega.4c06483 (PMC11780438; doi:10.1021/acsomega.4c06483)
Supplement: Supplementary file 1 — ao4c06483_si_001.pdf [file ao4c06483_si_001.pdf]

Tunable magnetic order in Fe-Mg codoped montmorillonite nanoclay interfaced with amino acids

Dinesh Thapa*[†,‡], Steven Westra[‡], Victoria Oas[‡], Dmitri Kilin[‡], and Svetlana Kilina*[‡]

[†] Department of Mathematics and Physics, Thomas More University, Crestview Hills, KY 41017, USA
[‡] Department of Chemistry and Biochemistry, North Dakota State University, ND 58108, USA

*To whom correspondence should be addressed: *thapad@thomasmore.edu, svetlana.kilina@ndsu.edu*

Supporting Information

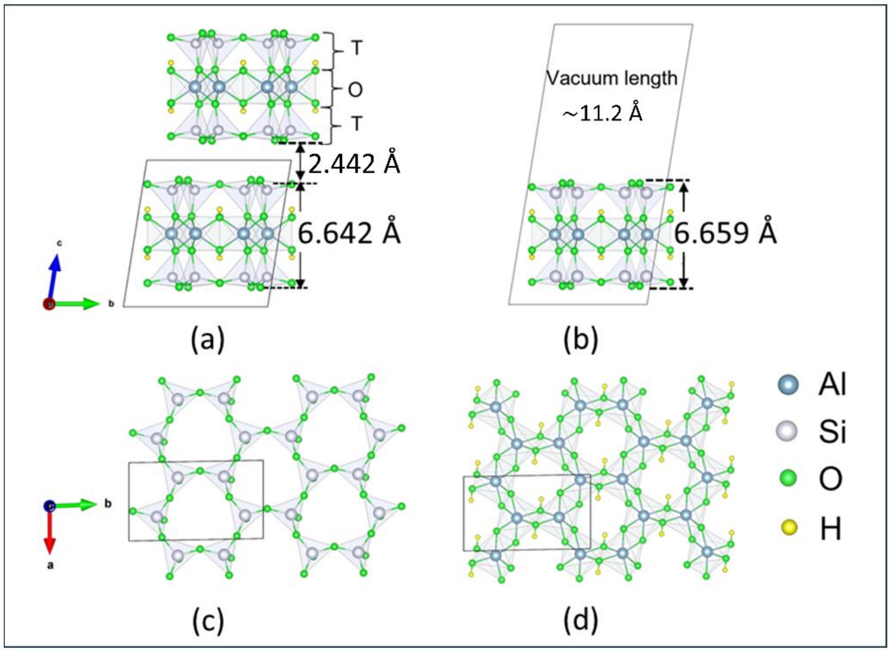

Fig. S1 Relaxed primitive geometrical structures of (a) pristine bulk MMT clay with Octahedral (O) sandwiched between two tetrahedral (T) sheets, (b) single layer nanoclay of MMT clay with vacuum length ~11.2 Å, (c) Top view of tetrahedral (T) sheet, and (d) Top view of octahedral (O) sheet. The black solid lines represent the size of the unit cells.

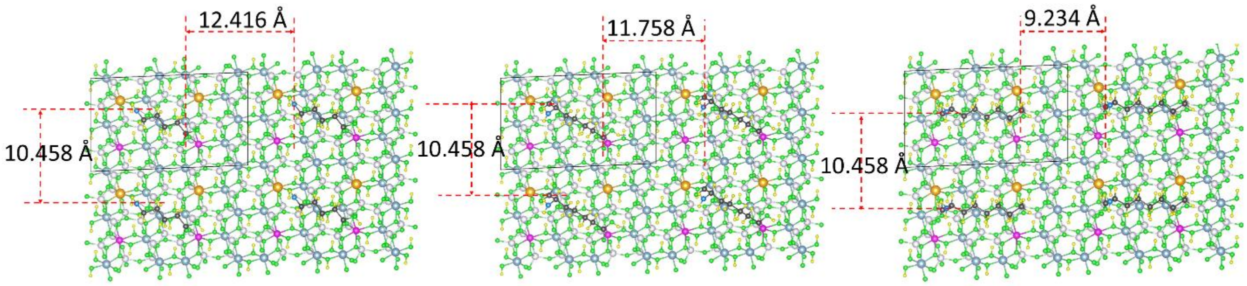

(a) Sa + AA1 (b) Sa + AA2 (c) Sa + AA3

Fig. S2 Diagram representing alignment of the three different amino acids AA1, AA2, and AA3, on the nanoclay model Sa as viewed from the top along (001) plane. The black solid line represents the size of the super cell. The carbon atoms on the amino acid chain are represented by black spheres. The distance between the chain of the amino acid is sufficiently large to reduce the interaction with its image. The alignment of three different amino acids is identical in remaining nanoclay models Sb, Sc1, and Sc2.

Table S1. Calculated value of the AA-nanoclay binding energy (E_b) in eV for the most stable magnetic order out of four magnetic configurations (AFM, FEM, FM, and NM) during the adsorption of amino acid molecules, AA1, AA2, and AA3 in each of the different nanoclay models Sa, Sb, Sc1, and Sc2 in vacuum and in aqueous medium (+51.H_2O) with their corresponding ground state magnetic order.

Slab	AA	In Vacuum	Ground state magnetic order	In aqueous medium	Ground state magnetic order
Sa	AA1	−1.617	FEM	−4.511	AFM
Sb		−1.895	AFM	−4.336	FM
Sc1		−1.538	FEM	−5.323	FM
Sc2		−2.089	FM	−5.824	FM
Sa	AA2	−1.916	FM	−6.228	AFM
Sb		−1.931	AFM	−5.803	FM
Sc1		−2.315	AFM	−6.486	FM
Sc2		−2.250	AFM	−6.381	FM
Sa	AA3	−1.758	FM	−5.017	AFM
Sb		−1.769	FM	−3.169	FM
Sc1		−2.119	AFM	−4.547	AFM
Sc2		−2.189	FM	−5.025	FM

Table S2. The minimum adsorption height (h_{min}) and the height of different atoms on AA1 molecule, nitrogen (h_N), carbon C1 (h_{C1}), carbon C5 (h_{C5}), oxygen O1 (h_{O1}), hydrogen H1 (h_{H1}), and hydrogen H2 (h_{H2}) due to adsorption of AA1 on the nanoclay model Sa, Sb, Sc1, and Sc2 in vacuum as well as in aqueous medium (+51.H_2O).

Slab	AA	h_{min} (Å)	h_N (Å)	h_{C1} (Å)	h_{C5} (Å)	h_{O1} (Å)	h_{H1} (Å)	h_{H2} (Å)
Sa	AA1	1.807	2.767	2.830	2.974	2.497	2.336	1.807
Sb		1.791	2.747	2.830	2.946	2.491	2.324	1.791
Sc1		1.839	2.825	2.823	2.974	2.466	2.304	1.839
Sc2		1.869	2.813	2.807	2.985	2.459	2.284	1.869
Sa	AA1+51.H_2O	1.874	3.148	3.285	2.941	2.577	2.840	2.464
Sb		1.877	3.136	3.293	2.939	2.576	2.836	2.444
Sc1		1.890	3.247	3.299	2.966	2.599	2.881	2.627
Sc2		1.810	3.394	3.247	2.940	2.520	2.816	2.818

Table S3. The minimum adsorption height (h_{min}) and the height of different atoms on AA2 molecule, nitrogen (h_N), carbon C1 (h_{C1}), carbon C7 (h_{C7}), oxygen O1 (h_{O1}), oxygen O2 (h_{O2}), hydrogen H1 (h_{H1}), and hydrogen H2 (h_{H2}) due to adsorption of AA2 on the nanoclay model Sa, Sb, Sc1, and Sc2 in vacuum as well as in aqueous medium (+51.H_2O).

Slab	AA	h_{min} (Å)	h_N (Å)	h_{C1} (Å)	h_{C7} (Å)	h_{O1} (Å)	h_{O2} (Å)	h_{H1} (Å)	h_{H2} (Å)
Sa	AA2	1.602	4.927	2.923	2.837	2.492	2.286	1.674	1.602
Sb		1.580	4.929	2.932	2.810	2.500	2.265	1.693	1.580
Sc1		1.592	4.922	2.949	2.821	2.520	2.292	1.727	1.592
Sc2		1.589	4.899	2.904	2.781	2.479	2.262	1.659	1.589
Sa	AA2+51.H_2O	1.781	4.400	2.915	2.867	2.574	3.291	2.267	3.065
Sb		1.781	4.388	2.914	2.865	2.573	3.297	2.267	3.069
Sc1		1.792	4.446	2.928	2.877	2.594	3.316	2.289	3.085
Sc2		1.764	4.410	2.902	2.844	2.562	3.258	2.256	3.037

Table S4. The minimum adsorption height (h_{min}) and the height of different atoms on AA3 molecule, nitrogen (h_N), carbon C1 (h_{C1}), carbon C8 (h_{C8}), oxygen O1 (h_{O1}), hydrogen H1 (h_{H1}), and hydrogen H2 (h_{H2}) due to adsorption of AA3 on the nanoclay model Sa, Sb, Sc1, and Sc2 in vacuum as well as in aqueous medium (+51.H_2O).

Slab	AA	h_{min} (Å)	h_N (Å)	h_{C1} (Å)	h_{C8} (Å)	h_{O1} (Å)	h_{H1} (Å)	h_{H2} (Å)
Sa	AA3	1.316	5.132	2.829	3.160	2.132	1.316	2.949
Sb		1.311	5.179	2.863	3.196	2.162	1.311	2.975
Sc1		1.343	5.139	2.851	3.134	2.165	1.343	2.914
Sc2		1.258	5.160	2.839	3.144	2.128	1.258	2.927
Sa	AA3+51.H_2O	1.911	5.012	2.822	3.754	2.337	1.911	3.904
Sb		1.259	4.916	2.842	3.592	2.017	1.259	3.733
Sc1		1.357	4.990	2.796	3.632	2.127	1.357	3.775
Sc2		1.668	4.998	2.779	3.713	2.276	1.668	3.875

Table S5 The charge transfer (oxidation state) of atoms in free nanoclay models Sa, Sb and Sc in vacuum before the adsorption of AA molecules. Here, O-clay represents the oxygen atom of the nanoclay models. The quantitative description of the charge transfer values among the atomic species was obtained using the Henkleman's Bader charge analysis program from the pre-converged total charge density. The total charge transfer on an atom, the oxidation state, was estimated by integrating the charge density within a Bader volume associated to each atomic species.

Atoms	Sa	Sb	Sc
Fe	+2.030	+2.029	+2.031
Mg	+2.000	+2.000	+2.000
Si	+4.005	+4.005	+4.005
O-clay	−1.960	−1.960	−1.959

Table S6. The charge transfer (oxidation state) of atoms in the most reactive AA-slab configurations in vacuum and in aqueous medium (+51.H_2O). Here O-clay and O-AA represent the oxygen atoms of the MMT nanoclay and amino acid molecules, respectively, and C1 is the first carbon atom of the amino acid chain according to the text notations. The calculations were done using the same method as described in Table S5.

Atoms	In Vacuum			In aqueous medium (+51.H_2O)		
	**Sc2+AA1**	**Sc1+AA2**	**Sc2+AA3**	**Sc2+AA1**	**Sc1+AA2**	**Sc2+AA3**
Fe	+2.020	+2.019	+2.019	+2.020	+2.021	+2.020
Mg	+2.000	+2.000	+2.000	+2.000	+2.000	+2.000
O-clay	−1.969	−1.969	−1.969	−1.979	−1.976	−1.979
O-AA	−1.844	−1.859	−1.937	−1.858	−1.874	−1.921
N	−2.500	−2.669	−2.671	−2.398	−2.248	−2.494
C1	+2.683	+2.896	+3.051	+2.702	+2.800	+2.917

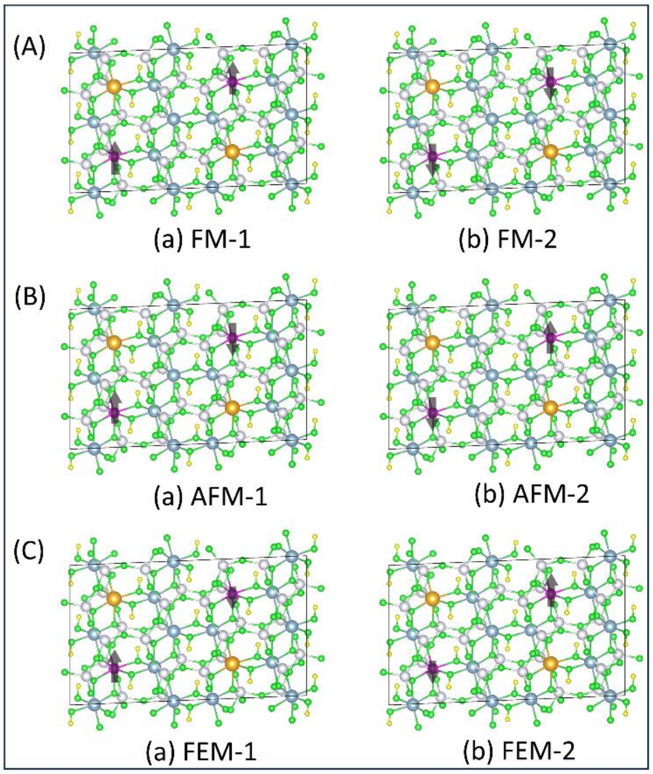

Fig. S3 Diagram illustrating different magnetic orders, (A) Ferromagnetism (FM), (B) Anti-ferromagnetism (AFM), and (C) Ferrimagnetism (FEM) due to Fe atoms with spin up (up-arrow) and spin down (down-arrow). The height of the arrow represents the magnitude of the magnetic moment associated with Fe atoms in the respective magnetic configurations.

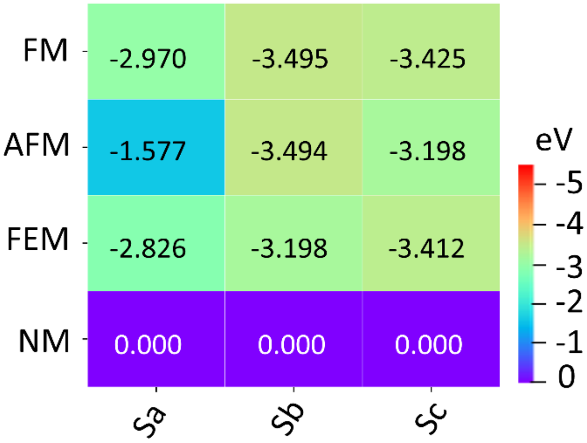

Fig. S4 Heat map representing magnetic order competition in three different free slab models before the adsorption of amino acids. Here, Sc represents both the periodically identical slabs Sc1 or Sc2. Here, the numbers in the inset represent relative energy values in eV of different magnetic orders FM, AFM, and FEM with respect to non-magnetic (NM) configuration which is referenced to zero.

Table S7. Comparison of the total magnetic moment (m_{tot}) per supercell ($2\times 2\times 1$), the local magnetic moment on each Fe (II) dopant (m_{Fe1} and m_{Fe2}) of the MMT nanoclay, and the most stable ground state magnetic order due to the adsorption of AA1 and AA2 molecules on the surface of nanoclay model Sa in vacuum using U_{eff} =3.0 eV and U_{eff} =4.0 eV. Here, the ground state magnetic order remains consistent for the U_{eff} values.

U_{eff} (eV)	Nanoclay Sa	Magnetic order	E_{tot} (eV)	m_{tot} (μ_B)	m_{Fe1} (μ_B)	m_{Fe2} (μ_B)	Ground state magnetic order
3.0	+AA1	AFM	-1049.6302	0.000	+2.854	-1.978	FEM
		FEM	-1050.1439	+2.143	+4.202	-1.979	
		FM	-1050.1394	+9.857	+4.080	+4.066	
	+AA2	AFM	-1082.9627	0.000	-1.979	+2.528	FM
		FEM	-1083.4637	+2.002	-1.982	+4.181	
		FM	-1083.4822	+7.908	+4.219	+1.979	
4.0	+AA1	AFM	-1048.5131	0.000	-4.238	+4.294	FEM
		FEM	-1049.2151	+3.907	-2.121	+4.300	
		FM	-1049.1459	+9.824	+4.301	+4.302	
	+AA2	AFM	-1082.8038	0.000	-4.297	+4.217	FM
		FEM	-1082.5447	+3.907	-2.123	+4.292	
		FM	-1082.8037	+10.011	+4.268	+4.233	
